# Utilizing Agro-Waste as Aggregate in Cement Composites: A Comprehensive Review of Properties, Global Trends, and Applications

**DOI:** 10.3390/ma18102195

**Published:** 2025-05-09

**Authors:** Ivanka Netinger Grubeša, Dunja Šamec, Sandra Juradin, Marijana Hadzima-Nyarko

**Affiliations:** 1 Department of Construction, University North, 42000 Varaždin, Croatia; 2 Department of Food Technology, University North, 48000 Koprivnica, Croatia; dsamec@unin.hr; 3 Faculty of Civil Engineering, Architecture and Geodesy, University of Split, 21000 Split, Croatia; sandra.juradin@gradst.hr; 4 Faculty of Civil Engineering and Architecture, Josip Juraj Strossmayer University of Osijek, 31000 Osijek, Croatia

**Keywords:** agro-waste, aggregates, cement composite, physical properties, mechanical properties, thermal properties, durability properties

## Abstract

Amid growing environmental concerns and the increasing demand for sustainable construction practices, the exploration of alternative materials in building applications has garnered significant attention. This paper provides a comprehensive review of the use of agricultural waste as an aggregate in cementitious composites, with a particular focus on palm kernel shells, coconut shells, hazelnut, peanut and pistachio shells, stone fruit shells and pits, date and grape seeds, rice husks, maize (corn) cobs, and sunflower seed shells. For each type of agro-waste, the paper discusses key physical and mechanical properties, global production volumes, and primary countries of origin. Furthermore, it offers an in-depth analysis of existing research on the incorporation of these materials into cement-based composites, highlighting both the advantages and limitations of their use. Although the integration of agro-waste into construction materials presents certain challenges, the vast quantities of agricultural residues generated globally underscore the urgency and potential of their reuse. In line with circular economy principles, this review advocates for the valorization of agro-waste through innovative and sustainable applications within the construction industry.

## 1. Introduction

Concrete, the most widely used construction material, primarily consists of cement, aggregate, and water. Aggregate constitutes the majority of concrete by volume, typically comprising between 60% and 80% of the total mixture [1]. In 2020, global concrete consumption was estimated at 26 Gt, necessitating the use of approximately 20 Gt of natural aggregates in concrete production [2,3]. Given the progressive depletion of natural aggregate resources, significant research efforts are being directed toward incorporating alternative materials, including industrial by-products and recycled constituents. The selection of materials throughout the construction process plays a pivotal role in determining the overall sustainability of the final structure. These environmental implications manifest directly through the avoidance of hazardous substances such as asbestos and indirectly via the integration of recycled materials and the reduction of landfill waste. Several industries, including plastics, steel, glass, construction, and automotive manufacturing, generate substantial amounts of by-products, many exhibiting potential as viable replacements for natural aggregates [4].

**Figure 1:**
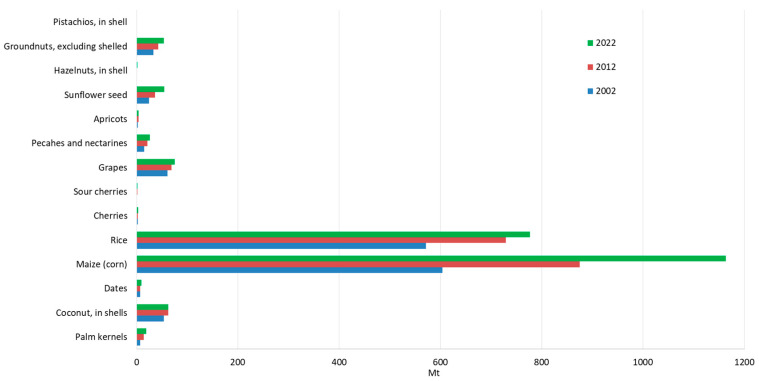
Production of some agricultural products in the period 2002–2012–2022; production data are taken from Food and Agriculture Organization of the United Nations (FAO) data on their website [10].

The United Nations has emphasised the need for global initiatives to reduce fossil fuel consumption, to cut greenhouse gas emissions, and to transition to a zero-waste economy [5,6]. Agriculture, responsible for approximately one-fifth of global greenhouse gas emissions [7], plays a significant role in this context. The rapid growth of agriculture and the increasing demand for agricultural products are exerting mounting pressure on the environment, ecosystems, and human health [5,6]. Consequently, using agricultural waste for alternative energy production is crucial for sustainable energy supply and green development [8]. Agricultural waste includes organic and inorganic residues generated from farm activities, such as crop cultivation, livestock farming, agro-industrial processing, and other related agricultural activities. Around 998 million tonnes of agricultural waste are generated yearly, contributing significantly to environmental pollution and contamination [9]. The production of widely used agricultural products in 2002, 2012, and 2022 is shown in Figure 1 in million tonnes (Mt).

The data presented in Figure 1 show a pronounced upward trend in the production of the observed agricultural products. Figure 1 also shows that the production of maize, sunflowers, and palm kernels more than doubled in the period analysed, indicating significant growth in these sectors. The total production volume of all agricultural products shown in Figure 1 for the year 2022 amounts to 2251.22 (Mt).

The increase in agricultural production is accompanied by a proportional rise in agricultural waste, thereby heightening the need for effective waste management solutions. The findings show that considerable amounts of agricultural waste are available, offering the possibility of using this waste as a substitute for traditional components in concrete production. If not adequately managed, agricultural waste is often destroyed or improperly disposed of in the environment, leading to significant environmental problems. Over time, the properties of this waste can change, leading to harmful and toxic effects on human health and ecosystems. Improper disposal methods, such as burning or dumping in landfills or open fields, release greenhouse gases such as carbon dioxide, methane, and nitrogen oxides [11]. These emissions pose a threat not only to public health but also to the environment. In addition, such practices contribute to soil and water contamination, exacerbating environmental problems and emphasising the need for sustainable waste management solutions [11].

Crop residues, including stalks, leaves, husks, kernels, corn cobs, and straws, are the by-products left over after harvesting or processing crops [12]. Given the significant amount of this waste generated in agriculture, modern industry increasingly seeks ways to reuse it, recognising its potential as a valuable renewable resource [13]. However, the high contents of lignin, cellulose, and hemicellulose in crop residues makes them tough, fibrous, and resistant to digestion by humans or animals, making them unsuitable for direct use as food or animal feed. In addition, some agricultural waste contains secondary metabolites such as tannins and alkaloids, limiting their use in the food industry. Given these limitations, considerable efforts are being made to utilise agricultural wastes in the non-food sector and convert them into valuable materials for sustainable applications [13]. One possible solution to the disposal problem of agricultural waste is its use in the construction industry as part of building materials in the form of binders/fillers or aggregates. Recent studies have shown the potential of agricultural waste in the concrete industry as a substitute for cement. Due to their high amorphous silica content, agro-industrial pozzolanic waste materials, such as rice husk ash, palm oil fuel ash, and bamboo leaf ash, have shown remarkable pozzolanic properties [14,15,16,17].

However, as aggregates comprise the largest volume fraction in concrete, using agricultural waste as an aggregate would be a more efficient approach. This article comprehensively overviews agricultural waste’s physical and mechanical properties, especially pits and shells, as potential concrete aggregates. Additionally, it presents the results of research that has investigated the physical, mechanical, and durability properties of concrete using agro-waste aggregate (AWA).

## 2. Properties of Agro-Waste Aggregate (AWA)

Before being used in concrete, each component, including aggregates, must undergo rigorous testing to determine its physical, mechanical, and other relevant properties. Table 1 summarises the properties of various agro-waste aggregates (AWAs) investigated in the literature for concrete production or other applications. For conventional concrete, properties such as specific gravity, loose and compacted bulk density, and water absorption are fundamental. It is important to note that some authors do not specify whether the reported bulk density refers to loose or compacted material; therefore, a dedicated bulk density column has been included in the table as well. In contrast, properties like impact and crushing or Los Angeles abrasion values are of interest for specialised concretes.

## 3. Production and Properties of Agro-Waste Aggregate Concrete (AWAC) and Examples of Its Application

### 3.1. Palm Kernel Shells

#### 3.1.1. Production and Properties of Palm Kernel Shells

**Figure 2:**
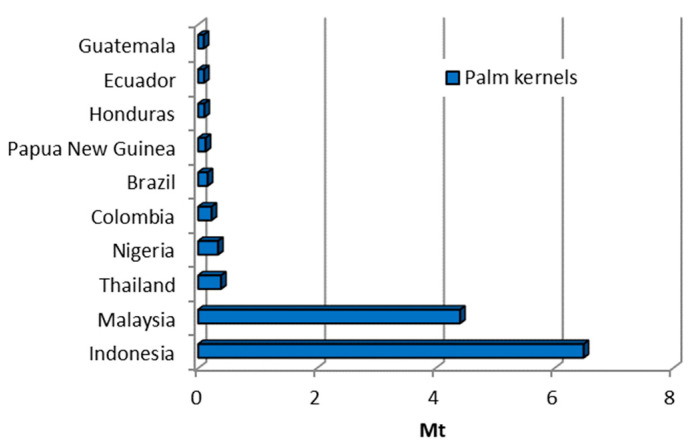
Production of palm kernels: Top 10 producers (average in period 2002–2022); the production data utilised in this study were retrieved from the website [10].

The oil palm (*Elaeis guineensis*) is a tropical tree plant known for its exceptionally high oil yield and extensive cultivation in tropical regions. As the world’s most crucial oil plant, it accounts for around 40% of all vegetable oil traded worldwide [47]. Palm oil is extracted from the fruit’s fleshy mesocarp (the pulp) and is used extensively for food, cosmetics, and biofuels [47]. Oil production also generates by-products such as palm kernel shells. It is expected that the growing demand for palm oil, fuelled by its increasing use in biodiesel, will lead to a significant increase in the production of palm kernel shells in the future. In the period under review, production has increased 2.66-fold, as shown in Figure 1. Figure 2 shows the ten largest producers of palm kernels worldwide from 2002 to 2022 based on FAO data [10]. Indonesia and Malaysia are the leading producers of palm kernel shells, producing around 11 million tonnes during the observed period.

The colour of palm kernel shells (PKSs) ranges from dark grey to black. Crushed shells typically have an angular or irregular shape, depending on how they were crushed. The shell’s surfaces (inside and outside) are smooth, while the crushed edges are rough. The most important physical and mechanical properties of PKS are summarised in Table 1. The specific gravity of PKS is significantly lower than that of conventional aggregates, such as crushed granite, which ranges from 2.6 to 2.7 [48], as shown in Table 1. According to Shafigh et al. [48], the PKS shell thickness varies from 0.5 mm to 8 mm, while the Los Angeles abrasion value is 3–5%, indicating good wear resistance. The water absorption capacity of PKS is significantly higher than that of conventional aggregates. The water absorption of PKS is between 8.15% and 12% after 1 h [48,49] and up to 33% [48] after 24 h compared to less than 1% for standard aggregates. This emphasises the significantly higher water absorption potential of PKS. Other notable properties are a flakiness index of 65% and an elongation index of 12% [50]. The impact value of the aggregate is between 2% and 8%, and the crushing value of the aggregate is between 0.2% and 10%. Both values are lower than normal aggregates, indicating that PKS has good impact absorption [48].

#### 3.1.2. Palm Kernel Shells as AWA

Due to the properties described above, palm kernel shells are a lightweight material suitable as an aggregate for lightweight concrete (LWC). In 1984, Abdullah [51] conducted the first investigation in Malaysia on the use of palm kernel shells as an AWA in lightweight concrete. The author successfully produced concrete with a compressive strength of up to 20 MPa by replacing the entire normal aggregate (granite) mass with palm kernel shells. Okafor [52] conducted experiments with six different mixes, varying the weight proportions of ordinary Portland cement, river sand, and coarse aggregate: granite (three mixes) and palm kernel shells (three mixes). The study showed that palm kernel shell concrete requires a higher proportion of fine aggregates than similar concrete with normal aggregates and can achieve a compressive strength of up to 30 MPa. In particular, the workability results showed that palm kernel shell concretes have better workability with the same free water content despite the irregular shape of the shells, which normally affects workability. The improvement in workability was attributed to the higher content of fine aggregate. Yew et al. [53] investigated the influence of original and crushed palm kernel shells of different types and age categories on workability. Their study revealed that increasing the age category of the palm kernel shells enhanced the workability of concrete. In their second paper [53], the authors subjected palm kernel shell aggregate to heat treatment for 0.5 and 1 h at two different temperatures (333.15 K and 423.15 K). They found that the workability of palm kernel shell concrete improved with increasing temperature and duration of heat treatment. Alengaram et al. [49] reported higher workability of palm kernel shell concrete compared to conventional concrete, noting that the palm kernel shell concrete had a higher superplasticiser content, higher cement content, and 10% silica fume and 5% fly ash by mass of cement. The authors compared the properties of oil palm kernel shell concrete (OPKSC) with regular-weight concrete (NWC). The results revealed that the strength development of OPKSC after 28 days was higher than that of NWC, probably due to the addition of silica fume.

**Figure 3:**
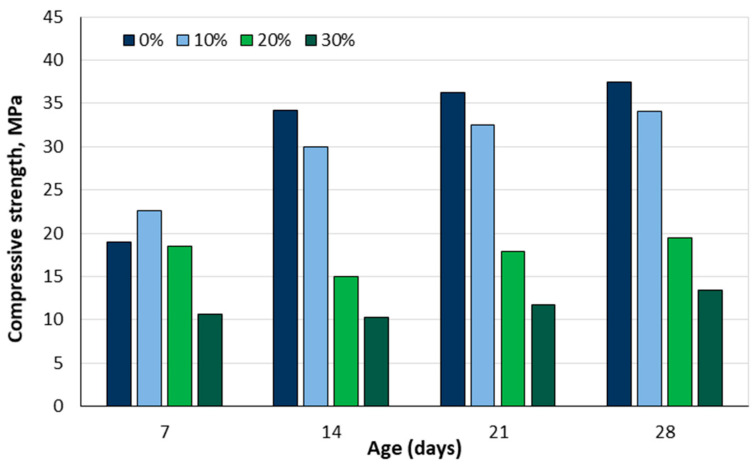
The results of the compressive strength obtained after 7 days, 14 days, 21 days, and 28 days of concrete curing with partial replacement of coarse aggregate volume for palm kernel shells [55].

Ndoke [54] conducted tests with asphalt concrete and concluded that palm kernel shells could be used as a partial substitute for coarse granite aggregate, up to 10% of mass for heavily trafficked roads and up to 50% of mass for lightly trafficked roads. Ifeanyi et al. [55] investigated the replacement of natural coarse aggregate with palm kernel shells at 0%, 10%, 20%, and 30% of volume. The results of the compressive strength tests are shown in Figure 3. The data show that the compressive strength decreases as the proportion of palm kernel shells in the concrete increases. Based on these results, the authors recommended that concrete with a 10% content is suitable for columns, beams, and foundations, while concrete with a 20% content could be suitable for suspended slabs.

Khankhaje et al. [56] presented a study on the development of sustainable, permeable, lightweight concrete in which natural coarse aggregate (size 6.30–9.50 mm) was partially replaced by AWA from the palm oil industry (size 4.75–6.30 mm and 6.30–9.50 mm) with a proportion of 25 to 75% by weight. Various properties were analysed in the study, including density in the fresh and cured state, void content, compressive and tensile strength, and permeability. The results indicate that sustainable, permeable, lightweight concrete can be produced using lightweight waste materials from the palm oil industry and that this type of concrete is suitable for use on low-traffic roads and parking lots. In addition, the permeable concrete with palm kernel shells showed high water permeability of 4 to 16 mm/s, with acceptable compressive strengths between 6 and 12 MPa.

**Figure 4:**
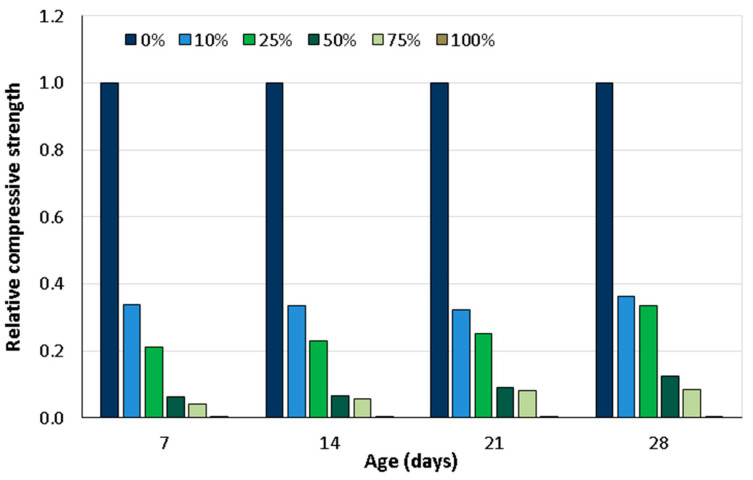
Relative compressive strength of concrete with partial replacement of coarse aggregate mass by palm kernel shell [57].

Mannan and Ganapathy [50] investigated the potential use of palm kernel shells as an AWA in concrete. The palm kernel shell aggregates used in their study were graded to a maximum size of 12.5 mm and accounted for about 40% of the total mix volume, representing a significant reduction in the use of conventional building materials, as palm kernel shells are solid waste products in manufacturing plants. For all curing methods applied to palm kernel shell concrete (Table 2), the compressive strength continued to develop over time but remained lower than that of normal concrete. Specifically, the compressive strength of palm kernel shell concrete was about 49–55% lower than that of conventional concrete. Azunna [57] recommended that replacing coarse aggregate with palm kernel shells should not exceed 25% of the total mass. In this study, the compressive strength of the concrete at 28 days was 4.78 MPa for the 10% mass replacement and 4.44 MPa for the 25% mass replacement, which did not meet the requirements for structural lightweight concrete. Compared to the reference concrete (0% palm kernel shells), which had a compressive strength of 13.22 MPa at 28 days, the reductions for concretes with 10% and 25% replacement of the natural aggregate with palm kernel shells were 63.84 and 66.41, respectively (Figure 4). Mixtures with a higher replacement percentage of 50%, 75%, and 100% gave significantly lower results with compressive strengths of 1.67 MPa, 1.11 MPa, and 0.0 MPa, respectively, which were lower than those reported in some other studies.

Serge et al. [58] conducted a study in which they used different proportions of palm kernel shells (PKSs) in volume fractions of aggregates (0%, 25%, 50%, 75%, and 100%). The results indicated that concrete with a 25% aggregate replacement achieved a compressive strength of 19.66 MPa after 28 days, whereas the reference concrete (0% PKS) had a compressive strength of 41.55 MPa. When replacing 100% of the natural aggregate with palm kernel shells, the compressive strength decreased significantly to around 5.37 MPa. However, with a 50% replacement, the concrete exhibited a significant reduction in mass, achieving a density of <2000 kg/m^3^, thereby qualifying as lightweight concrete.

The compressive strength of concrete mixtures containing palm kernel shells as a partial or complete replacement for conventional aggregates varies greatly. It depends on factors such as the composition of the mix, the percentage of replacement, the curing methods, the origin and quality of the palm kernel shells, the particle size, and the treatment of the shells. Table 2 shows the results of various studies. In general, the compressive strength of lightweight concrete with palm kernel shells as an aggregate substitute, with or without cementitious materials, is in the typical range for structural lightweight concrete (20–35 MPa), with a density about 20–25% lower than that of conventional concrete. However, recent studies have shown that palm kernel shells can be used to produce high-quality lightweight concrete, achieving compressive strengths of up to 53 and 56 MPa after 28 and 56 days, respectively [59]. Shafigh et al. [59] proposed a novel method for producing high-strength lightweight concrete from solid agricultural waste, particularly by crushing large old palm kernel shells. This method demonstrated that concrete in which palm kernel shells replaced 100% of the coarse aggregate achieved compressive strength comparable to that of conventional concrete, encouraging the construction industry to adopt this environmentally friendly option for real-world applications [48].

Mo et al. [60] improved the performance of concrete containing palm kernel shells as an AWA and ground blast furnace slag (GGBS) as a partial cement replacement by incorporating low-volume steel fibres. Their experimental results indicated that increasing the GGBS replacement led to a deterioration of the concrete properties, whereas adding low-volume steel fibres improved the mechanical properties, stress–strain behaviour, and flexural toughness. The best results were obtained with a mixture of 20% GGBS as partial cement replacement and 0.6% steel fibres (Table 2).

### 3.2. Coconut Shells

#### 3.2.1. Production and Properties of Coconut Shells

**Figure 5:**
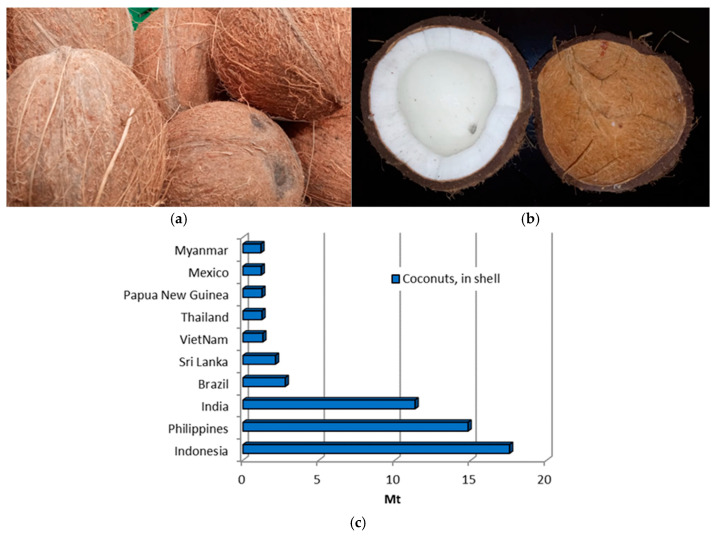
Coconuts (**a**) and cross-section of coconut (**b**). Production of coconuts (**c**), in shell. Top 10 producers (average in period 2002–2022); production data were taken from the website [10].

Coconut (*Cocos nucifera* L.) (Figure 5a,b) is cultivated in over 85 countries worldwide, with annual production exceeding 62 million tonnes. In 2022, Asia accounted for 85.1% of the global coconut crop, with Indonesia, the Philippines, and India being the largest producers, as shown in Figure 5c [10].

The coconut fruit consists of three distinct layers: the exocarp (a glossy, smooth outer skin or husk), the mesocarp (a fibrous husk), and the endocarp (a hard, woody shell) [65], as illustrated in Figure 5b. The endocarp, typically brown and hard, is a protective layer for the coconut kernel. The coconut husk is often discarded as agricultural waste following nut processing [18]. Some important physical and mechanical properties of coconut shells are summarised in Table 1. Coconut shells have a thickness of 0.15 mm to 8 mm [19,48] and are rougher than palm kernel shells. The specific gravity of coconut shells ranges from 1.05 to 1.74 [18], with a compacted bulk density of about 650 kg/m^3^ [48], which is more than twice as low as that of crushed aggregates. This low density makes coconut shells a promising material for lightweight concrete applications. Regarding physical properties, the 24-hour water absorption of coconut shells is 24%, the aggregate crushing value is 2.58%, and the aggregate impact value is 8.15% [48]. The Los Angeles abrasion value for coconut shells is 1.63%, which is significantly lower than the 24% observed for crushed granite [48]. Compared to palm kernel shells, coconut shells have a rougher surface, which can positively affect the compressive strength of concrete when used as an AWA.

#### 3.2.2. Coconut Shells as AWA

Coconut shells, a by-product of the coconut industry, are a sustainable alternative for concrete production. Due to their higher water absorption compared to conventional aggregates (Table 1), coconut shells absorb some of the water in the mix, which can reduce the effective water–cement ratio. If this is not considered, it can result in a stiffer mix, so additional water or admixtures may be required to achieve the desired workability. Coconut shell concrete generally shows less segregation than standard concrete. A review of the existing literature highlights that the biological decomposition of coconut shell concrete is not observed even after 365 days, as emphasised by various researchers in their studies [66].

**Figure 6:**
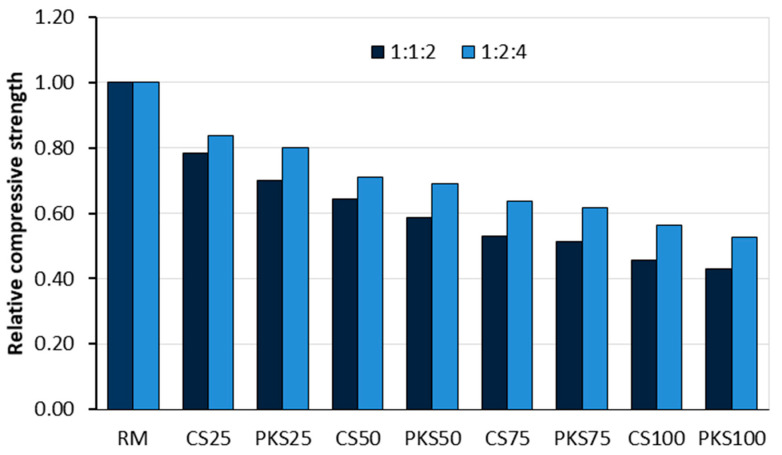
The results of the relative compressive strength at 28 days of curing for concrete with partial replacement of coarse aggregate volume by coconut shell and palm kernel shell [61].

The review of Eziefula [18] emphasises the potential of using coconut shells as a coarse aggregate in concrete mixes. In particular, the study demonstrated that replacing 18.5% of conventional crushed granite volume with coconut shells resulted in concrete suitable for reinforced structures. In addition, some experiments have shown that concrete made with 100% coconut shells can achieve compressive strengths exceeding 15 MPa, meeting the minimum standards set by the British Standards Institution. Yerramala and Ramachandrudu [67] compared the properties of concrete in which 0, 10, 15, and 20% of the coarse granite aggregate mass was replaced by coconut shell aggregate. A constant water–cement ratio of 0.6 was maintained for all concretes. The findings revealed that the density of the concrete decreased as the proportion of coconut shells increased. The workability decreased with increasing coconut shell content. The compressive and tensile strengths of the coconut shell concrete were lower than the same properties of the control concrete. The pores in the concrete and the absorption were higher in concretes with coconut shell substitutes than in the control concrete. They also concluded that even after 90 days of testing, the concrete with coconut shell aggregate consistently underperformed conventional concrete with granite aggregate. Olanipekun et al. [61] presented the results of a study that investigated strength properties of concrete made using crushed aggregate, granulated coconut shell (CS), and palm kernel shell (PKS) as a replacement for conventional coarse aggregate volume in proportions of 0% (RM—reference mix), 25% (CS25 and PKS25), 50% (CS50 and PKS50), 75% (CS75 and PKS75), and 100% (CS100 and PKS100). The study evaluated two mix ratios (1:1:2 and 1:2:4). In both mix ratios, concrete with coconut shells showed a higher compressive strength than palm kernel shell mixes. It was concluded that coconut shells are more suitable than palm kernel shells as substitutes for conventional aggregates in concrete production (Figure 6 and Table 3). Figure 6 illustrates the relative compressive strength values in relation to the reference/control mix. The compressive strength of the RM was 35 MPa for the 1:1:2 mix ratio and 27.5 MPa for the 1:2:4 mix ratio.

**Figure 7:**
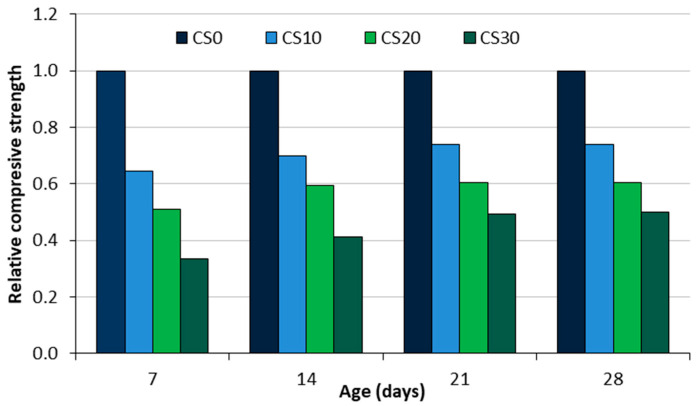
Relative compressive strength of concrete with partial replacement of aggregate mass by coconut shells for different ages: 7, 14, 21, and 28 days [71].

Gunasekaran and Kumar [68] investigated the properties of fresh concrete, including density and 28-day compressive strength, using coconut shells as a substitute for coarse aggregate in lightweight concrete. They concluded that crushed coconut shells are suitable for producing lightweight concrete, with an average fresh concrete density of 1975 kg/m^3^ and a 28-day compressive strength of 19.1 MPa. Osei [69] investigated concrete’s density and strength properties by replacing crushed granite with coconut shells. The results showed that the density and compressive strength decreased as the replacement percentage increased. Concrete with 20%, 30%, 40%, 50%, and 100% replacement by volume achieved 28-day compressive strengths of 19.7 MPa, 18.68 MPa, 17.57 MPa, 16.65 MPa, and 9.29 MPa, respectively, corresponding to 94%, 89%, 85%, 79.6%, and 44.4% of the strength of the reference concrete. This indicates that coconut shells can be used as a substitute for conventional aggregates in both conventional and lightweight reinforced concrete structures. Kanojia and Jain [70] found that replacing 40% of the conventional coarse aggregate volume with coconut shells resulted in a 62.6% reduction in compressive strength after 7 days but only 21.5% after 28 days. This replacement also made the concrete 7.47% lighter. Azunna et al. [71] investigated the effect of coconut shell as a replacement for fine and coarse aggregate in concrete in mass fractions of 10% (CS10), 20% (CS20), and 30% (CS30), respectively. The reference concrete, CS0, was without coconut shells. The compressive, flexural, and tensile strengths; density; and water absorption of dried concrete samples were evaluated after 7, 14, 21, and 28 days. The results showed that increasing the volume fraction of coconut shell replacement increased the workability and water absorption of the mixtures but decreased the 28-day compressive strength of the concrete by 24.11% (CS10), 39.60% (CS20), and 50.14% (CS30) compared to the reference concrete (CS0), as illustrated in Figure 7.

The mechanical properties of concrete containing coconut shells as a substitute for aggregates, as described in the scientific literature, are summarised in Table 3. While some studies suggested a complete replacement of coarse aggregate with coconut shells, most authors opted for a replacement percentage of up to 30%. Increasing the percentage of coconut shells as a substitute for coarse aggregate usually leads to a decrease in compressive strength. Although coconut shell concrete has a lower density and workability, which may require adjustment of the water content or admixtures, it is well suited for lightweight and non-structural applications.

### 3.3. Hazelnut, Peanuts, and Pistachios Shells

#### 3.3.1. Production and Properties of Hazelnut, Peanuts, and Pistachios Shells

Hazelnuts (*Corylus avellana*), peanuts (*Arachis hypogaea*), and pistachios (*Pistacia vera*) share several commonalities in terms of the waste generated during their processing. A significant portion of the harvested biomass often consists of shells or husks, which are frequently disposed of as waste. Although usually considered a by-product, these shells or husks represent a significant portion of the total biomass and pose a challenge to the environment if not managed properly.

**Figure 8:**
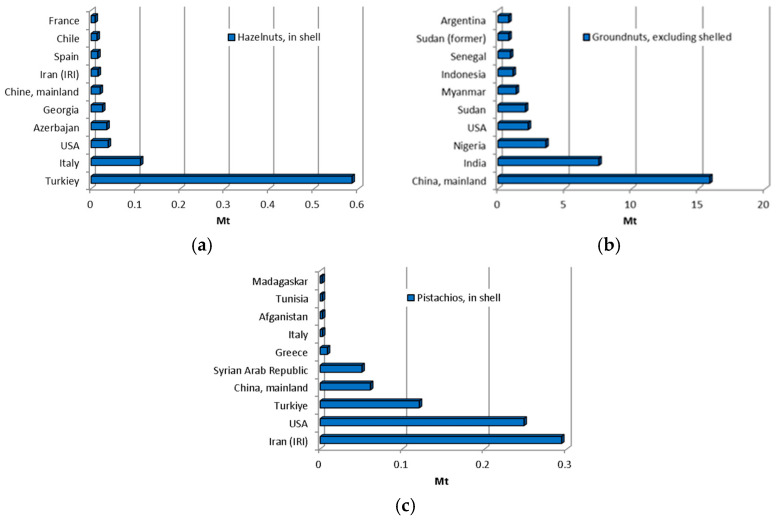
Production of (**a**) hazelnuts, in the shell; (**b)** groundnuts, excluding shelled; (**c**) pistachios, in the shell. Top 10 producers (average in period 2002–2022); production data were taken from the website [10].

In 2022, global hazelnut production (in shells) reached 1.2 million tonnes [10]. As shown in Figure 8a, the leading producers are Turkey (0.58 Mt), Italy, the United States, and Azerbaijan, with Turkey contributing around 65% of global production. After harvesting, hazelnuts are usually dried for several months to improve their chemical and physical stability and prevent mould growth. This drying process can take more than two years. When the hazelnuts are sent for industrial processing, they are usually shelled, which produces a significant amount of hazelnut shells as a by-product [42]. Hazelnut shells account for around 42% of the total biomass and are often considered waste material. However, they can be utilised as a raw material for the furfural industry or as biofuel for heating purposes. The composition of hazelnut shells includes hemicellulose (25–30%), cellulose (26–32%), lignin (40–43%), and other H_2_O-soluble extractives (3.3–4%) [76]. Hazelnut shell moisture content is around 14.38% [31], and the bulk density is 550.50 kg/m^3^ [42] (Table 1). The density is between 1.05 and 1.3 g/cm^3^, while the shell dimensions vary: length between 18.8 and 25.9 mm, width between 17.2 and 22.7 mm, and thickness between 1.3 and 1.7 mm [76]. According to Milošević and Milošević [33], the shell thickness can vary considerably between the different hazelnut genotypes, ranging from 0.95 to 2.41 mm (Table 1).

**Figure 9:**
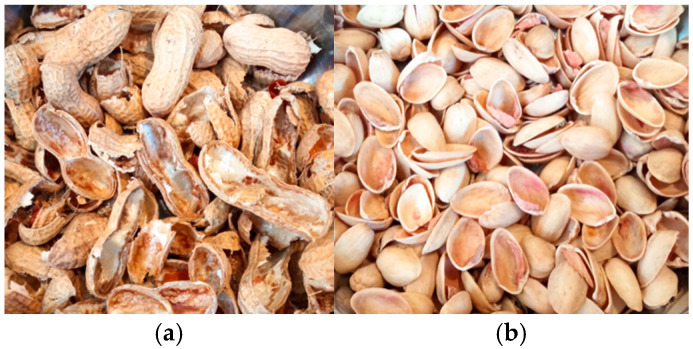
(**a**) Groundnut (peanut) shells; (**b**) pistachio shells.

Groundnuts, commonly referred to as peanuts, are mainly grown for oil extraction, direct consumption, and as animal feed. In 2022, global groundnut production was around 55 million tonnes, with China (15.83 Mt), India (7.54 Mt), and Nigeria (3.58 Mt) among the leading producers (Figure 8b). The extraction of peanut kernels produces peanut shells as a by-product (Figure 9a), with global production estimated at approximately 11 million tonnes annually. These shells are often underutilised and frequently disposed of by burning or natural decomposition. The composition of groundnut shells includes hemicellulose (62%), cellulose (18.10%), lignin (5.60%), and ash (4.70%) [77]. They account for approximately 30% of the total mass of the legume. When properly managed, groundnut shells can be repurposed as bioenergy feedstock, biocomposite materials, or as organic soil improvers, providing an opportunity to reduce (agricultural) waste and improve sustainability. Table 1 reports the bulk density of groundnut shells as 254.55 kg/m^3^ [30]. The same authors found that the water absorption of groundnut shells is 1.61%, making this material suitable for use as a fine aggregate in concrete under normal construction conditions. Jerzak et al. [31] reported a moisture content of 6.39% for peanut shells [24], as documented in Table 1.

In 2022, global pistachio production reached 1.03 million tonnes, with Turkey, the United States, and Iran being the largest producers (Figure 8c). The pistachio shell, which is naturally beige and remarkably tough for a plant shell (Figure 9b), constitutes a large portion of the fruit. Pistachio shells comprise 20–32% hemicellulose, 30–55% cellulose, and 12–38% lignin [78]. On average, the kernel content of pistachios is between 31.5% and 49.5%, while pistachio shells account for around 35% to 45% of the total waste generated in the pistachio industry [79]. This extensive biomass could be utilised for various applications, e.g., bioenergy production, biocomposites, or as a source of lignocellulosic materials contributing to sustainable waste management. Table 1 documents the specific gravity of pistachio shells as 0.12 [29], alongside a moisture content of 4.04% and a density of 1.43 g/cm^3^ [28]. Ryšavý et al. [77] used pistachio shells in pellet form and found that the bulk density of a mixture consisting entirely of pistachio shells was 286.9 kg/m^3^. In contrast, the bulk density of pistachio nuts is between 573 and 649 kg/m^3^ [80].

#### 3.3.2. Hazelnut Shells, Groundnuts (Peanut) Shells, and Pistachio Shells as AWA

**Figure 10:**
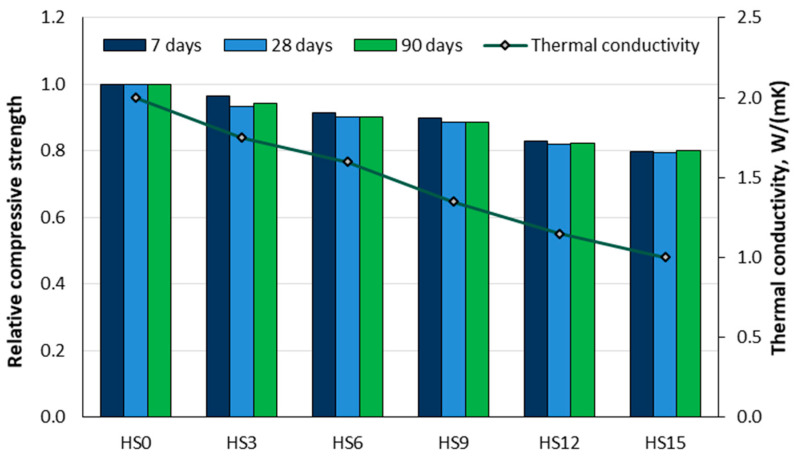
Relative compressive strength at 7, 28, and 90 days (primary vertical axis) and thermal conductivity (secondary vertical axis) of concretes with partial replacement of coarse aggregate mass by hazelnut shells [81].

Ozocak and Sisman [81] investigated the use of hazelnut shells by preparing concrete samples in which 3% (HS3), 6% (HS6), 9% (HS9), 12% (HS12), and 15% (HS15) of the coarse aggregate mass was replaced by hazelnut shells. They evaluated the physical, mechanical, and thermal properties of these concretes. As shown in Figure 10, the relative compressive strength decreased by 3.3% to 20.3% after 7 days of curing, by 6% to 20.9% after 28 days, and by 5.5% to 20.3% after 90 days. The figure also illustrates the thermal conductivity, with a maximum reduction of around 51% observed in the concrete with 15% hazelnut shells (HS15). The study concludes that hazelnut shells can be used as an aggregate in concrete production and enable the production of lightweight concrete with sufficient strength and durability, as well as improved thermal and acoustic insulation, provided that the proportion of hazelnut shells in the aggregate does not exceed 10%.

The suitability of groundnut shells as a constituent material in concrete was investigated by replacing part of the volume of fine aggregate (river sand) with groundnut shells [37]. Using peanut shells in concrete offers multiple benefits, as highlighted by Sada et al. [37]. These include improved waste management, cost efficiency, pollution reduction, and economic benefits for farmers by creating a market for this by-product, which, when sold, promotes increased agricultural production.

**Figure 11:**
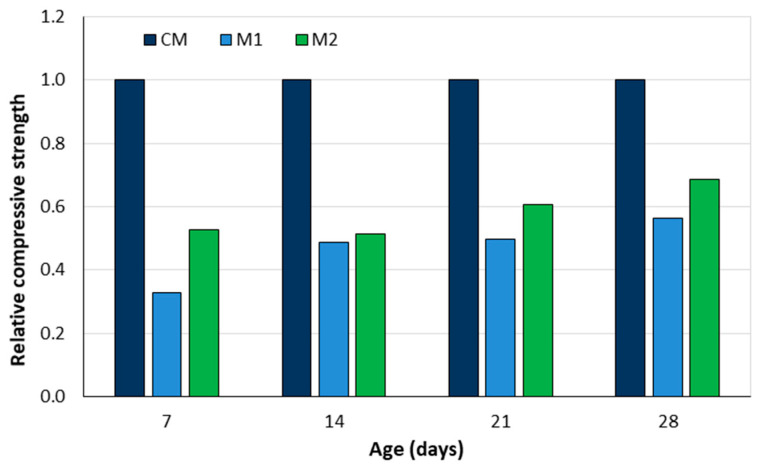
Relative compressive strength of concrete with partial replacement of fine aggregate mass by groundnut shell at 7, 14, 21, and 28 days [38].

However, due to high water absorption, incorporating peanut shells into concrete reduces its workability. As expected, the concrete’s density and compressive strength decrease as the proportion of peanut shells increases. Concrete with peanut shells can be used for non-load-bearing applications such as partition walls, floor slabs, and noise barriers. Research suggests prewetting the peanut shells to improve the workability of the concrete, but it should not be used in areas exposed to water, as moisture will affect its weight and strength [37]. Gandhare [38] also used groundnut shells for the partial replacement of sand. Three mixes were tested: a control concrete (CM) and two concretes with sugarcane bagasse ash as a partial replacement for cement (2% and 5% of the mass of cement) and groundnut shells (5% of the mass of the fine aggregate). The combination of sugarcane bag ash and groundnut shells reduced the workability with a slump of 55 mm and 40 mm for M1 and M2, respectively, compared to 90 mm for the control concrete. The compressive strength of the control concrete was 22.01 MPa (7 days), 26.89 MPa (15 days), 30.63 MPa (21 days), and 32.07 MPa (28 days). The relative compressive strengths are shown in Figure 11. While the combination of sugarcane bagasse ash and groundnut shells reduced the compressive strength, concretes with higher sugarcane bagasse ash content exhibited better strength at all ages, making them suitable for non-load-bearing slabs.

Islam et al. [80] investigated the effects of pistachio shells as a coarse aggregate in concrete using volume ratios of 0, 1, 2, and 4% pistachio shells. The workability of fresh concrete decreased significantly with increasing volumes of pistachio shells. The compressive strength decreased by 25%, 36%, and 58% compared to the control concrete when pistachio shells replaced 1%, 2%, and 4% of the coarse aggregate. The splitting tensile strength also decreased with increasing proportions of pistachio shells. The authors recommended an optimal pistachio shell dosage of 1% for concrete, as this substitution level minimises the adverse effects on concrete properties.

**Figure 12:**
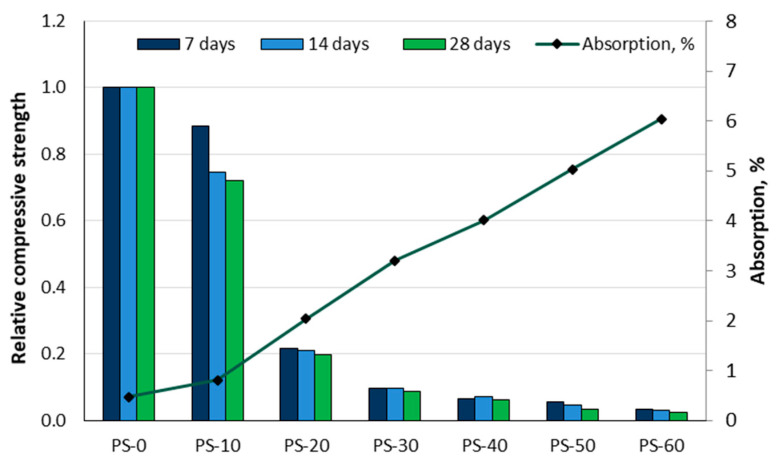
Relative compressive strength at 7, 14, and 28 days (primary vertical axis) and absorption (secondary vertical axis) of concrete with partial replacement of fine aggregate mass with pistachio shell [29].

Alsalami [29] used pistachio shells to partially replace sand in amounts of 10, 20, 30, 40, 50, and 60% by mass of fine aggregate and tested the compressive strength, density, and absorption results. Figure 12 illustrates the results for the relative compressive strength in relation to the control mixture and absorption. For the control mix (PS-0), the compressive strength was 31.5 MPa after 7 days, 42.14 MPa after 14 days, and 71.47 MPa after 28 days. While the compressive strength increased with age for all samples, it decreased with increasing proportions of pistachio shells. The water absorption ranged from 0.47% to 6.04%, increasing with a higher proportion of pistachio shells.

It was found that the density decreased as the replacement levels increased: from 2.316 g/cm^3^ for the control mixture (PS-0) to 1.21 g/cm^3^ (PS-50) and 1 g/cm^3^ (PS-60). Based on the findings, mixtures containing up to a 20% replacement ratio are deemed viable for structural load-bearing purposes, and mixtures with a 30% replacement ratio can be applied for non-load-bearing purposes. In comparison, mixtures with 50% and 60% replacement ratios achieved the density required for lightweight mortar.

### 3.4. Stone Fruit Shells and Pits

#### 3.4.1. Production and Properties of Stone Fruit Shells and Pits

Stone fruits (*Prunus* sp.) are a type of fruit characterised by a large, hard pit or stone in the centre that encloses the seed. This centre is surrounded by fleshy fruit, which makes it easy to distinguish stone fruit from other types of fruit. The scientific term for stone fruit is drupe and refers to the structure of the fruit, which typically consists of three layers: the outer skin (exocarp), the fleshy middle (mesocarp), and the hard pit or stone (endocarp) that houses the seed.

**Figure 13:**
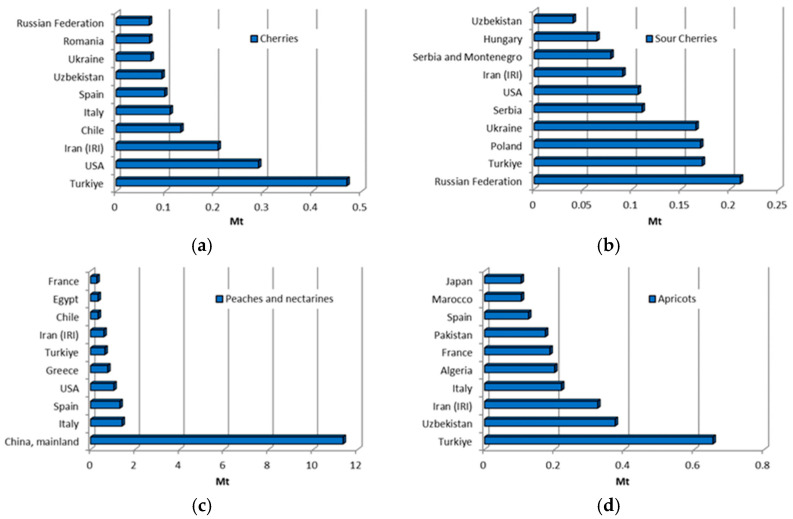
Production of (**a**) cherries; (**b**) sour cherries; (**c**) peaches and nectarines; (**d**) apricots. Top 10 producers (average in period 2002–2022); production data were taken from the website [10].

Cherries (*Prunus* sp.) are small stone fruits belonging to the genus Prunus, with two main types being sweet cherry (*P. avium*) and sour cherry (*P. cerasus*). The main difference between the two lies in their flavour, with sweet cherries being notably sweeter and usually eaten fresh, while sour cherries are tart and are often used in processed products such as jams, cakes, and juices. The estimated total global production of cherries and sour cherries for 2022 was over 4 million tonnes (Figure 13a,b) [11]. The four largest producers—Russia, Turkey, Poland, and Ukraine—account for over half of the total production. In contrast, in 2022, the ten largest sour-cherry-producing countries accounted for approximately 90.5% of the global sour cherry harvest. In 2022, global sour cherry production reached 1.59 million tonnes, marking a 63.9% increase compared to two decades ago (Figure 1). In the same year, the global harvest of cherries reached 2.79 million tonnes, with Turkey leading in production and contributing over 19% of the total output. Cherries are originally native to Asia but are now cultivated extensively in temperate regions, including the Mediterranean.

**Figure 14:**
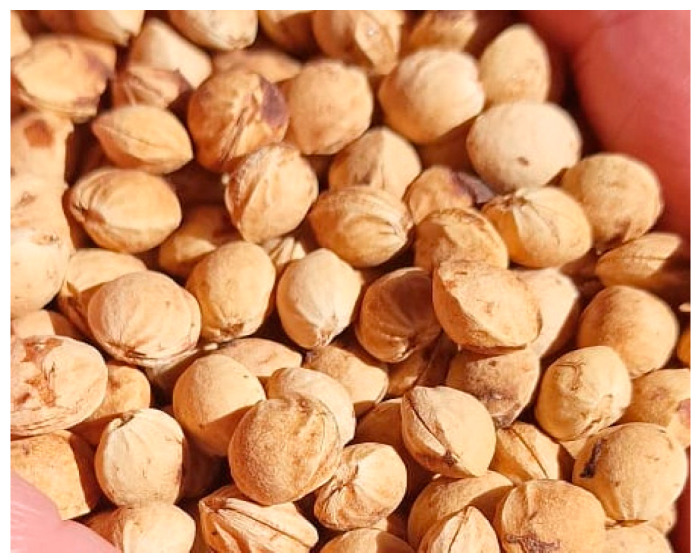
Cherry pits.

Every year, large quantities of fruit seeds, particularly pits (Figure 14), are discarded during the production of juices, jams, pies, and other processed products. These residues can constitute up to 60% of the total weight of the fruit and are often discarded, resulting in significant losses for producers [11]. Cherry pits represent about 14.6% of the total mass of cherries [82]. According to [83], the average length of the cherry pit ranges from 9.78 to 11.03 mm, the width is between 7.83 and 9.50 mm, and the weight varies from 0.27 to 0.39 grammes. The ratio of pulp to stone is between 17.70 and 20.73. The density of cherry pits is 813 kg/m^3^ [26], and the bulk density is 472.88 kg/m^3^ [34], as shown in Table 1.

Peach and apricot (*P. persica*) stone fruits are very popular worldwide, and their production has grown significantly over the last two decades. This growth is characterised by an increase of more than 78%, from 14.82 million tonnes in 2002 to 26.35 million tonnes in 2022 (Figure 1). Figure 13c shows the top 10 peach-producing countries from 2002 to 2022, with China dominating global peach production with a share of around 70%. Global peach production generates considerable waste, consisting mainly of peel, pits, and pomace. A peach fruit consists of three main parts: the mesocarp (pulp), the exocarp (peel), and the endocarp (stone or pit), which contains the seed in a shell. The seed constitutes about 6% of the endocarp, while the shell comprises about 94% of the mass. As detailed in Table 1, peach shells have a specific gravity of 1.26 [20,33,36] to 1.28 [29], which is notably lower than conventional coarse aggregates [4] or river sand [20,33,36]. The impact value of peach shells is 1.95 [33,36], and the Los Angeles (LA) abrasion value is around 6% [20,33], both of which are lower than those for typical aggregates [4], for impact values from 22.54 [33] to 24 [4], for LA abrasion. These properties position peach shells as a promising alternative material for diverse applications, including concrete production.

Similar to peaches and nectarines, the production of apricots (*P. armeniaca*) increased by almost 50% between 2002 and 2022 (Figure 1). According to Figure 13d, Turkey, Uzbekistan, and Iran dominated global apricot production in 2022, collectively accounting for 1,560,195 tonnes. With such a large production output, thousands of tonnes of apricot shells are produced as waste, which are often disposed of as garbage or incinerated. However, apricot peels are increasingly processed for diverse industries. Ground apricot shells are actively used for sandblasting, surface cleaning, in the chemical industry, paint removal, polishing, and as an abrasive in metal and furniture manufacturing, as well as in military facilities and aircraft maintenance [84]. The most important physical and mechanical properties of apricot shells are summarised in Table 1. Apricot shells’ specific gravity ranges from 1.42 [27] to 1.44 [36]. According to [36], the apricot shell has a strength of 8.5 MPa, which is greater than the value of a peach shell (3.4 MPa). Wu et al. [40] described that crushed peach and apricot shells have a flaky shape, while river sand is rounded [27], with the inside of the shells being smooth. D’Eusanio et al. [36] further described the surface of apricot shells as rough, irregular, and with many voids. The flakiness index for peach and apricot shells is between 41 and 48 and 48 and 56, respectively [27]. These properties make apricot shells a potential material for various applications, including construction and industry.

There is currently no organised system for collecting and recycling stone fruit pits, leading to potentially losing valuable resources [85,86]. However, small quantities of these pits are utilized as heating fuel, in biodiesel production, or as fillers in cement manufacturing [82].

#### 3.4.2. Stone Fruit Shells and Pits as AWA

**Figure 15:**
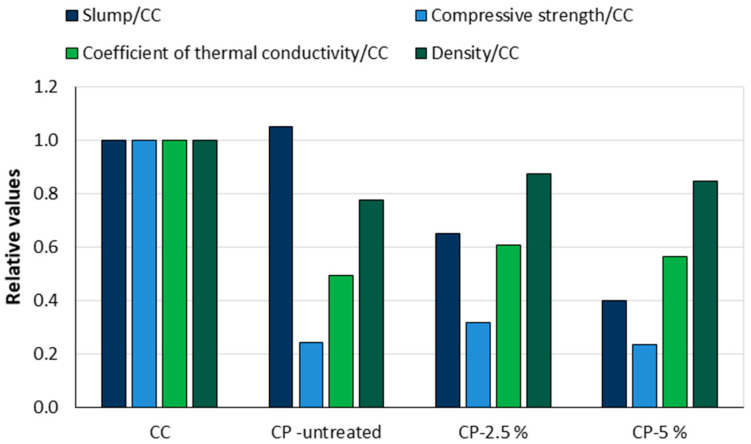
Relative values of workability (slump), compressive strength, coefficient of thermal conductivity, and density of concrete with complete replacement of coarse aggregate volume by cherry pits [34].

Netinger Grubeša et al. [34] investigated the influence of the complete replacement of coarse dolomite aggregates (4/8 mm fraction) by untreated and treated cherry pits on fresh and hardened concrete properties. The cherry pits were treated with 2.5% and 5% NaOH solution. The effect of replacing the coarse aggregate with cherry pits is shown in Figure 15 by the ratio of the properties of the concrete mixes and the control concrete mix (CC). The control concrete had a density of 2365.62 kg/m^3^, a compressive strength of 61.98 MPa, a thermal conductivity coefficient of 1.42 W/mK, and a slump of 20 cm. Mixtures with cherry pits, treated (CP-2.5% and CP-5%) and untreated (CP-untreated), had lower density, lower thermal conductivity coefficient, and significantly lower compressive strength results. The workability of mixtures with treated cherry pits decreased proportionally to the concentration of the alkaline solution. It was observed that the treatment of the cores significantly increased the water absorption.

**Figure 16:**
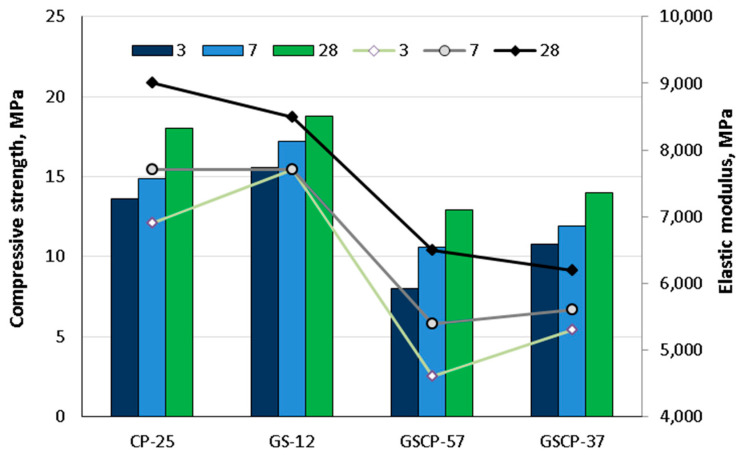
Compressive (primary vertical axis) and elastic modulus (secondary vertical axis) of concrete with partial replacement of aggregate volume by cherry pits and grape seeds [83].

Zwicky [83] tested concrete with Portland cement, untreated sawdust, and mineral and organic aggregates. The mineral aggregates were expanded clay, expanded glass, sand, and gravel, while the organic aggregates were cherry pits (CPs) and grape seeds (GSs). The results are presented here for mixtures with 25% (CP-25), 12% (GS-12), 57% (GSCP-57), and 37% (GSCP-37) organic aggregates. The author’s markings indicate that organic aggregates were incorporated into concrete mixes. Figure 16 shows the compressive strength and modulus of elasticity for the selected mixtures. The findings demonstrate that all evaluated properties exhibited a significant effect regarding the organic aggregate content. Furthermore, the strength development with organic additives is deemed sufficient to meet industrial production requirements.

The annual disposal of peach shell waste in China exceeds one thousand tonnes. Peach shells’ lightweight and regenerative properties compared to normal-weight aggregates make them a promising option for lightweight aggregates and sustainable building materials in producing lightweight concrete.

Wu et al. [87] have shown that using peach shells instead of normal-weight aggregates positively affects concrete density and that a reduction in density of up to 30% can be achieved using peach shells in concrete. However, peach shell concrete’s splitting tensile strength, flexural strength, and modulus of elasticity are lower than those of lightweight concrete made from other lightweight aggregates such as pumice and expanded clay. The low tensile strength typically results in significant tensile cracking under tensile loading conditions. Consequently, enhancing the mechanical properties of peach shell concrete demands further investigation. Fibre reinforcement represents an effective method for enhancing concrete’s mechanical properties, particularly in terms of the splitting tensile strength and flexural strength. Wu et al. [27] tested crushed peach shell concrete, crushed apricot shell concrete, and carbonised peach and apricot shell concrete to determine the effect of carbonation on the physical, mechanical, and triaxial creep properties of lightweight aggregate concrete. The results showed that replacing raw aggregates (crushed peach and apricot shells) with carbonized aggregates reduced the density, water absorption, and open porosity and increased the overall porosity of lightweight concrete. The mechanical properties were also significantly improved. The mixture with carbonized apricot shells achieved the highest compressive strength, splitting tensile strength, flexural strength, and modulus of elasticity, which were 44.2%, 55.3%, 54.6%, and 42.5% higher, respectively, than the same properties of a concrete mixture with crushed apricot shells.

In a subsequent study, Wu et al. [40] tested a series of specimens in which 12.5%, 25%, 37.5%, and 50% of the conventional aggregate volume was replaced with a mixture of crushed peach and apricot, maintaining all other parameters as constants. The results showed that when 50% of the normal aggregate and sand were replaced with peach and apricot shells, the 28-day compressive strength, splitting tensile strength, flexural strength, and modulus of elasticity of the concrete decreased by 34.3%, 28.8%, 33.6%, and 31.5%, respectively. However, the concrete containing 50% of a mixture of peach and apricot shells had a 19.37% lower density and thus met the density requirements for concrete with lightweight aggregates. In addition, the water absorption and porosity of the concrete increased as the proportion of the peach and apricot skin mixture increased. Based on the results, using a mixture of peach shells and apricot shells as coarse and fine aggregates to produce lightweight concrete with acceptable properties seems to be a viable option. In [43], Wu et al. prepared a series of mixtures of 25%, 50%, 75%, and 100% peach shells as substitutes for the normal weight aggregate volume, keeping the other parameters constant. The results revealed that a full replacement (100%) of normal-weight aggregate with peach shells resulted in reductions of 44.0%, 34.5%, 46.5%, and 43.4% in compressive strength, splitting tensile strength, flexural strength, and modulus of elasticity, respectively. Additionally, the 24-hour water absorption and porosity increased to 10.2% and 17.4%, respectively. They concluded that concrete containing no more than 50% peach shells has adequate mechanical properties for lightweight concrete.

**Figure 17:**
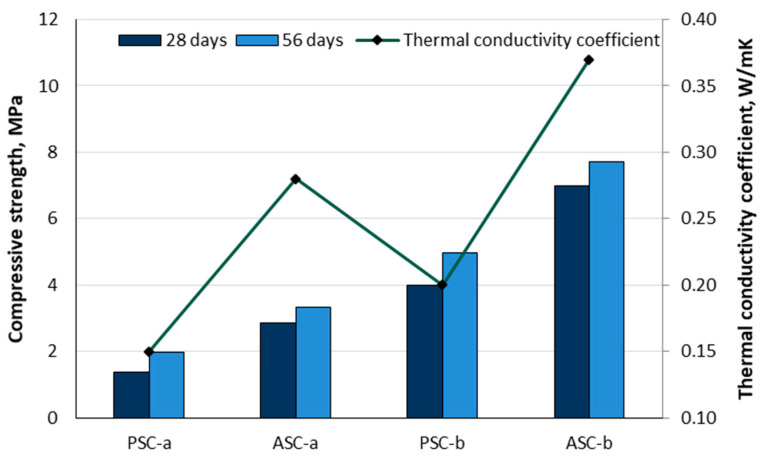
Compressive strength values at 28-day and 56-day (primary vertical axis) and the thermal conductivity coefficient (secondary vertical axis) of concrete with peach and apricot shells as aggregates [36].

In non-structural lightweight concrete, D’Eusanio et al. [36] replaced coarse aggregates with peach and apricot shells. The fruit pits were dried and crushed to a 4.5–9.5 mm particle size. Two types of binders were used: lime only (mark a) and a combination of lime and cement (mark b). Peach shell samples (PSCs) exhibited lower density (1000−1200 kg/m^3^) due to their highly porous structure, whereas apricot shell samples (ASCs) achieved higher values in the range of 1120 to 1260 kg/m^3^. Figure 17 presents the compressive strength values measured at 28 and 56 days, along with the thermal conductivity coefficient of such lightweight concrete. PSC samples have a lower thermal conductivity (0.15–0.20 W/mK) and a lower compressive strength (1–4 MPa). The apricot shell is practically free of porosity and has a denser structure, so the ASC samples had the highest values for thermal conductivity (0.2–0.4 W/mK) and compressive strength (2.8–7.0 MPa).

**Figure 18:**
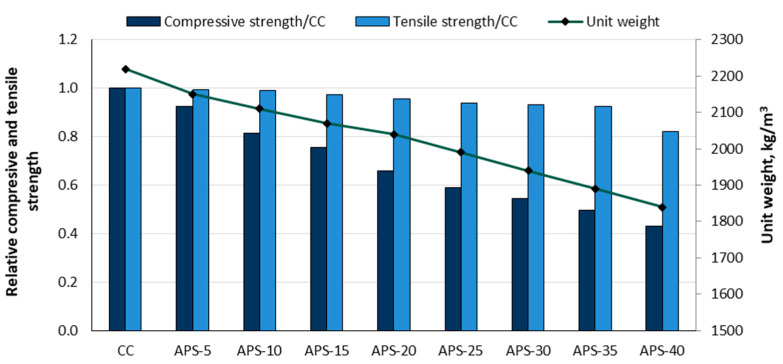
Relative compressive and tensile strength values (primary vertical axis) and unit weight (secondary vertical axis) of concrete with partial replacement of aggregate volume by apricot shell aggregate [88].

Yildiz et al. [88] replaced fine (0–4 mm) and coarse (4–8 mm) limestone aggregate volume with different percentages (5, 10, 15, 20, 25, 30, 35, and 40%) of the apricot shell (APS). A control concrete (CC) was produced with 30 MPa and a mix ratio of cement/water/gravel/sand = 1/0.55/1.28/2.38 by weight. The control concrete achieved a compressive strength of 34.65 MPa, a tensile strength of 4.17 MPa, and a unit weight of 2220 kg/m^3^. The relative values of compressive and tensile strength compared to the control concrete for the APS-containing concrete and the unit weight are shown in Figure 18. The unit weight of the APS concrete was gradually reduced from 3.15% to 17.12% as the APS content increased. Compressive strength reductions ranged from 7.50% to 56.94%, while the tensile strength decreased by 0.48–17.75%. APS concrete is not recommended for structural elements but can be used for many other elements, such as pavements, partition walls, paving stones, etc.

The summary of tests on the potential use of stone fruit shells and pits—specifically cherry pits, peach shells, and apricot shells—as aggregates in concrete is presented in Table 4.

### 3.5. Date Seeds

#### 3.5.1. Production and Properties of Date Seeds

**Figure 19:**
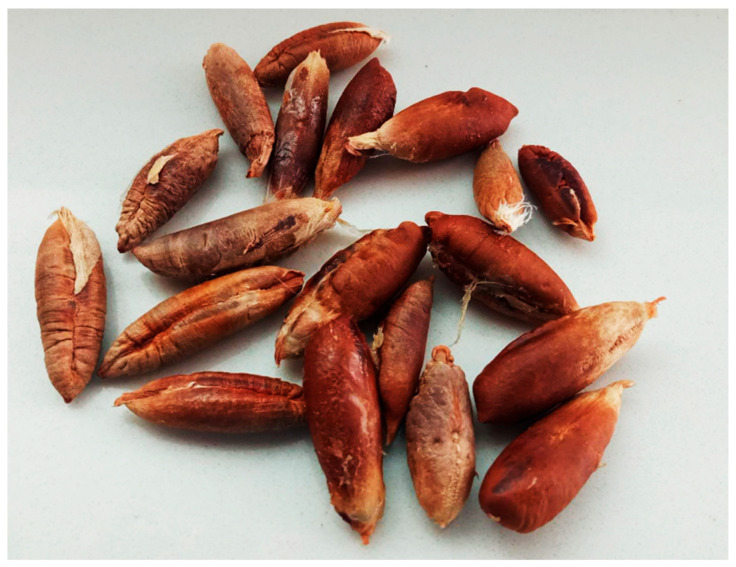
Date palm seed.

Date seeds are a by-product of date consumption, mainly from the date palm (*Phoenix dactylifera* L.), which grows in regions such as the Canary Islands, North Africa, the Middle East, Pakistan, India, and California [89]. Despite their nutritional value and widespread consumption by humans and animals [89], date seeds (Figure 19) are often discarded, contributing to landfill waste and posing a challenge to the environment.

**Figure 20:**
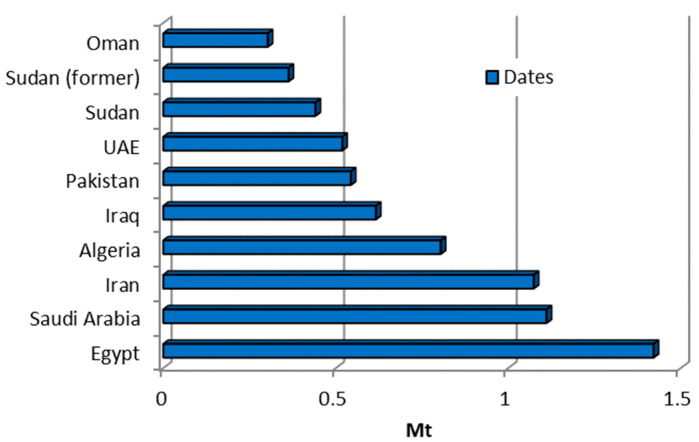
Production of dates. Top 10 producers (average in period 2002–2022); production data were taken from the website [10].

The date palm can reach a height of up to 23 m and is characterised by a stem marked by the cut-off remains of old leaves. It culminates in a crown of elegant, shiny, pinnate leaves that can grow up to 5 m long. The date fruit, which is actually a berry, contains a single elongated seed and varies considerably in shape, size, colour, quality, and consistency of the pulp depending on the growing conditions. A single bunch of dates can contain over 1000 fruits weighing 8 kg or more. The dried fruit consists of more than 50% sugar and around 2% each of protein, fat, and minerals [89]. As shown in Table 1, the water absorption capacity of date seeds ranges from 8.1% [20] to 36% [39]. Their specific gravity lies between 1.13 [39] and 1.39 [20], their impact value varies from 6.83% [39] to 22% [20], and their ratio of compacted bulk density to loose bulk density is 1.14 [19,39]. Figure 20 shows the ten countries with the highest date production, with Egypt, Saudi Arabia, and Iran as the three largest producers at the top. These countries collectively contributed approximately half of the global date production during the analysed timeframe. Total date fruit production has shown steady growth, rising from 6.72 million tonnes in 2002 to 9.75 million tonnes in 2022 [10].

#### 3.5.2. Date Seeds as AWA

**Figure 21:**
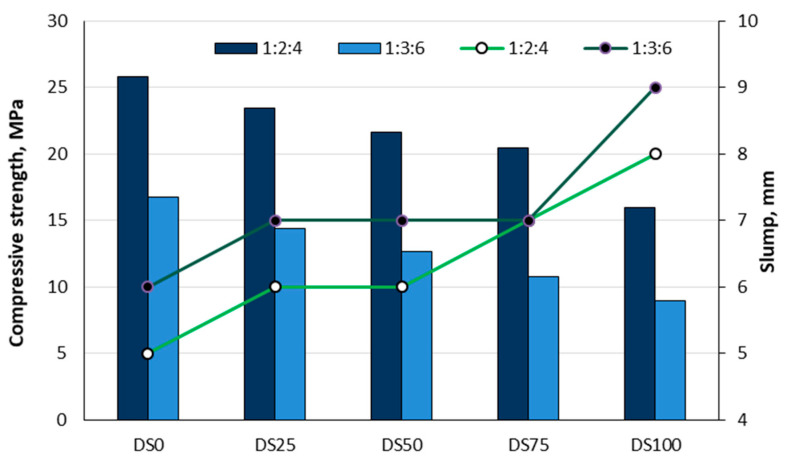
Compressive strength (primary vertical axis) and workability—slump (secondary vertical axis)—of concrete with partial replacement of coarse aggregate mass by date seeds [20].

The potential use of date seeds (DSs) in concrete production could not only alleviate the problem of agricultural waste in societies but also significantly reduce the cost of concrete production. Adefemi et al. [20] conducted a study to investigate whether date seeds have comparable properties to crushed granite, how the replacement of crushed granite with different proportions of date seeds affects the compressive strength of hardened concrete, and to what extent the inclusion of date seeds could reduce the cost of concrete production. They tested concrete mixtures with ratios of 1:2:4 and 1:3:6. Concrete cubes measuring 150 × 150 × 150 mm^3^ were cast with different mass percentages of crushed granite to date seed as coarse aggregate: 100:0 (DS0), 75:25 (DS25), 50:50 (DS50), 25:75 (DS75), and 0:100 (DS100). The slump test results indicated that the incorporation of date seeds did not substantially influence concrete workability. The compressive strength tests revealed that partial replacement of crushed granite with date seeds (DSs) in concrete mixtures (ratios 1:2:4 and 1:3:6) met compressive strength requirements, except at 100% replacement. For example, the reduction in compressive strength was about 9.15% to 37.89% for the 1:2:4 mix and 14.18% to 46.36% for the 1:3:6 mix (Figure 21). The study concluded that date seeds can be partially used as a substitute for coarse aggregate in producing lightweight concrete and can be recommended as an alternative material (partial replacement) for coarse aggregate [20].

The aim of the study conducted by Ahmed et al. [90] was to investigate the strength behaviour of concrete containing date seeds exposed to a sodium chloride (NaCl) and sodium sulphate (Na_2_SO_4_) solution. Date seeds replaced the coarse aggregate in 2, 3, and 4% weight percentages. The water–cement ratio was constant at 0.5 in all mixtures. Workability was tested on fresh concrete, and density and compressive strength were tested on hardened concrete specimens cured in plain water and in a mixed solution of NaCl and Na_2_SO_4_ for 7 and 28 days. The experimental findings indicate that the data seeds did not significantly affect the workability of concrete mixtures. The density and compressive strength of the specimen cured in a mixed solution of NaCl and Na_2_SO_4_ were higher than the same properties of concrete cured in plain water. Palh et al. [91] investigated the use of date seeds as a replacement for coarse aggregate in concrete at replacement levels of 2%, 3%, and 4% by volume. The design mix ratio was 1:2:4 (cement: fine aggregate: coarse aggregate). The workability and compressive strength of the concrete decreased with increasing amounts of date seeds. Since the addition of date seeds reduced the compressive strength of the concrete, the authors in [91] suggested using date seeds in the concrete for the parts of the structure for which low or medium compressive strength is required.

**Figure 22:**
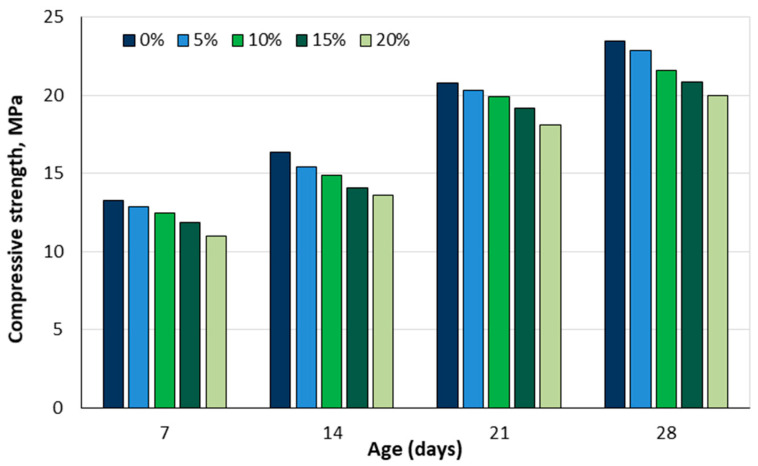
Development of compressive strength in concrete with partial replacement of coarse aggregate mass by date seed [93].

Yusuf et al. [92] tested concrete with date seeds as a substitute for coarse aggregates in percentages of 5, 10, 15, and 20% by mass. The concrete mixtures had a water–cement ratio of 0.5. A slump test was carried out to assess the workability of the fresh concrete. The freshly produced concrete was poured into 150 × 150 × 150 mm^3^ cube moulds, and the compressive strength was determined after curing in water for 7, 14, 21, and 28 days. The results showed that the concrete slump increased with increasing date seed content. The compressive strength was inversely proportional to the date seed content; concrete with 20% date seed replacing the coarse aggregate had the lowest compressive strength (20 MPa) (Figure 22).

Sarathkumar et al. [94] investigated the mechanical properties of using a mixture of date and tamarind seeds as a partial substitute for fine aggregates. These were washed, dried, and sieved to obtain medium-sized sand. The mixes were prepared by partially substituting fine aggregate with 10 to 20% date seed and 1.5 to 2.5% tamarind seed in M25-grade concrete mixes. Mechanical properties such as compressive, tensile, and flexural strength were evaluated using standardised cubes, cylinders, and prisms for each concrete mix. The mixture with a replacement of 10% date seed and 1.5% tamarind seed achieved a strength closer to that of the control concrete, while a further replacement reduced the strength of the concrete.

Table 5 summarises the findings from the previously described studies. In most of these studies, the percentage of substitutes did not exceed 20%, and the compressive strength was expected to decrease as the percentage of date seeds replacing the coarse aggregates increased.

### 3.6. Grape Seeds

#### 3.6.1. Production and Properties of Grape Seeds

**Figure 23:**
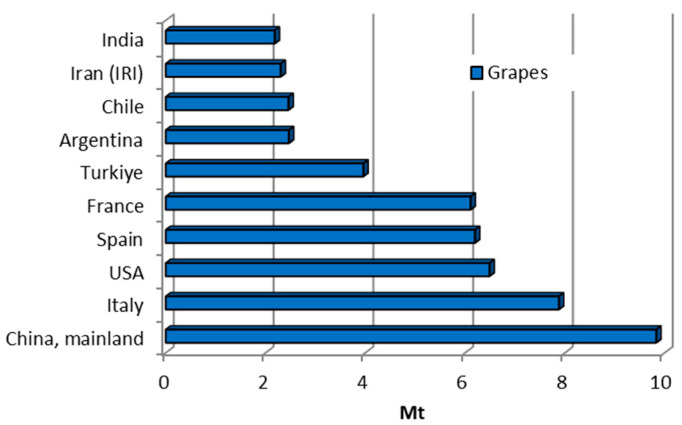
Production of grapes. Top 10 producers (average in period 2002–2022); production data were taken from the website [10].

Grape (*Vitis vinifera*) production holds substantial global economic importance worldwide due to its widespread cultivation for various uses, including fresh consumption, wine production, raisins, juices, and other products. With an annual production of about one million tonnes, the wine processing industry generates considerable waste, amounting to 13.5–14.5% of total production. Figure 1 and Figure 23 show global grape production and the leading producers. The main waste components of grapes are pomace, seeds, and stems [34]. Approximately 75% of 1000 kg of grapes are processed into wine, leaving 25% remaining as pomace [10].

**Figure 24:**
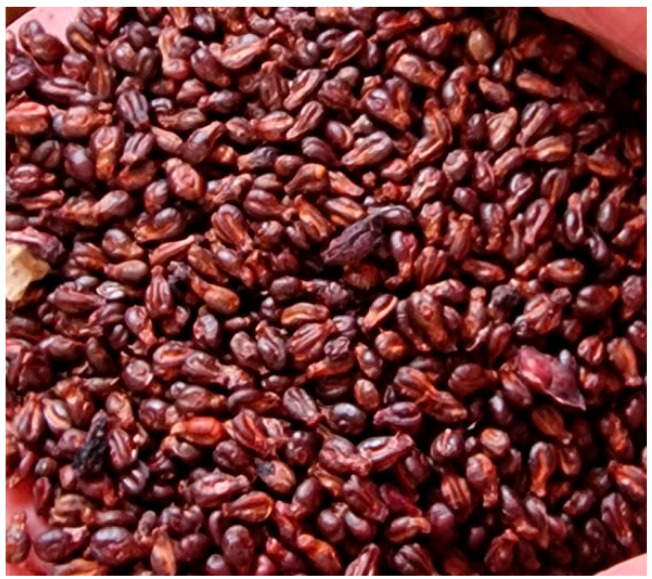
Grape seeds.

Grape pomace, a by-product of grape pressing, consists of bunches (if not removed), skins, seeds, and other residues [95]. Grape seeds comprise around 20–26% of the pomace, depending on the grape variety [96]. The global annual production of grape marc is estimated at approximately 9 million tonnes, with Croatia contributing around 40,600 tonnes to this total [97]. The pomace consists mainly of skins and pulp (51%), seeds (47%), and stalks (2%) [93]. Grape seeds, as shown in Figure 24, typically comprise 2–5% of the total grape mass and contribute to about 38–52% of the solid waste from the wine industry [98]. The bulk density of grape pomace ranges between 469.3 and 546.3 kg/m^3^, while porosity ranges from 52.87% to 55.67%, with both parameters being modulated by moisture content (12.26–24.61%). These values are summarized in Table 1. The density of grape seeds is about 1000 kg/m^3^ [34].

#### 3.6.2. Grape Seeds as AWA

**Figure 25:**
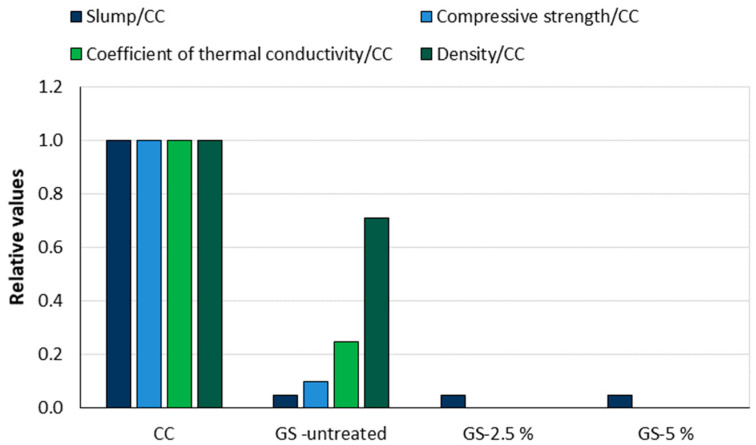
Relative values of workability (slump), compressive strength, coefficient of thermal conductivity, and density of concrete with complete replacement of fine aggregate volume by grape seeds [34].

In [34], grape seeds—both untreated and pretreated with 2.5% or 5% NaOH solution —were used as complete replacements for fine dolomite aggregate volumes in the concrete mixtures. Replacing the fine aggregate with grape seeds reduced the workability of the concrete from 20 cm for control concrete (CC) to 1 cm for mixtures with treated and untreated grape seeds. The compressive strength, density, and thermal conductivity coefficient were only measured on control concrete and untreated GS concrete, as the test specimens of the mixtures with treated grape seeds (GS-2.5% and GS-5%) probably disintegrated due to the high tannin content in the grape seeds. The relative results of the compressive strength, density, and thermal conductivity coefficient values are shown in Figure 25. Concrete with untreated grape seeds achieved 10% of the compressive strength (6.23 MPa), 25% of the thermal conductivity coefficient (0.36 W/mK), and 71% of the density (1674.64 kg/m^3^) of the control concrete. The findings align with those for cherry pits in the same study: Grape seeds significantly reduce workability and compressive strength but positively affect the concrete’s thermal properties. Furthermore, the incorporation of grape seeds in concrete mixes positively affects the reduction of concrete density, which may improve earthquake resistance in structures/buildings using this material.

### 3.7. Maize (Corn) Cobs

#### 3.7.1. Production and Properties

**Figure 26:**
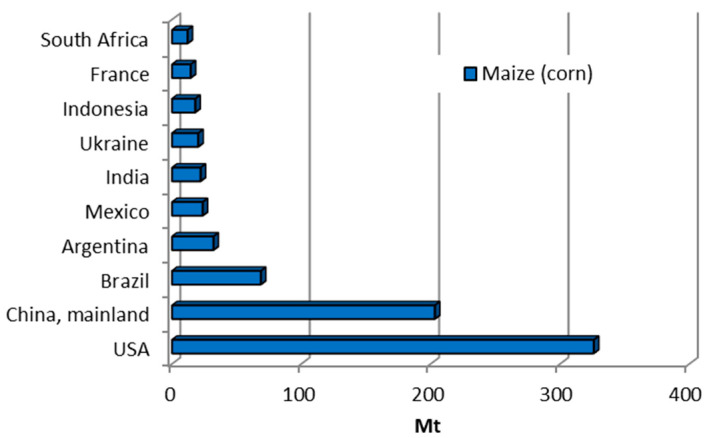
Production of maize (corn). Top 10 producers (average in period 2002–2022); production data were taken from the website [10].

Corn (*Zea mays*), also known as maize, is one of the most significant cereal crops in the world. It is a staple food in many countries and is crucial in producing animal feed, biofuels, and industrial applications. Corn cobs are the agricultural by-products that remain after the removal of corn grains. For each kilogram of dry corn kernels produced, approximately 0.15 kg of corn cobs is generated as a by-product [22]. Global corn production is dominated by the United States (contributing more than 43%), followed by Asia (30.3%) and Africa (7.4%), as shown in Figure 26 [10,18].

In 2022, global maize production reached 1162.1 Mt [10]. The biochemical composition of corn cobs comprises 39.1% cellulose, 42.1% hemicellulose, 9.1% lignin, 1.7% protein, and 1.2% ash [99]. The main inorganic component is oxygen (77.52%), together with silica (10.06%), aluminium (4.44%), potassium (2.2%), calcium (2.09%), magnesium (1.49%), sodium (1.14%), and iron (1.06%) [99]. Due to their high oxygen content, corn cobs have excellent thermal insulation properties. Pinto et al. [21] concluded that a board made from shredded corn cobs has a thermal conductivity comparable to expanded polystyrene. Corn cobs have an average density of about 212 kg/m^3^, making them suitable as an aggregate for lightweight concrete, as highlighted in Table 1. Their porosity ranges from 46.54% [23] to 67.93% [22]. In addition, corn cobs have a high water absorption capacity. Pinto et al. [21] conducted water absorption tests on ten randomly selected cobs, reporting values ranging from 258% to 374%. The specific gravity of corn cobs is 0.957 [23], and the moisture content ranges from 4.51% [23] to 6.38% [22] (Table 1).

#### 3.7.2. Maize (Corn) Cobs as AWA

Pinto et al. [100] investigated the use of crushed corn cob as a lightweight aggregate in non-structural concrete. The density, compressive strength, and thermal insulation properties of lightweight concrete with crushed corn cobs were tested. The results were compared with those of concrete with expanded clay. Concrete with crushed corn cobs showed better thermal insulation, which is an essential insulating property of lightweight aggregates. In addition, concrete with crushed corn cobs had a lower density and a significantly lower compressive strength than concrete with expanded clay as an aggregate. Faustino et al. [101] investigated the potential use of recycled corn cobs as an alternative to lightweight aggregate (LWA) for lightweight concrete (LWC) masonry blocks. The properties of corn cobs as a material were improved by coating them with cement paste. It was found that adding cement paste improved the adhesion between the concrete and the aggregate. The corn cob concrete demonstrated satisfactory durability under aggressive thermo-hygrometric cycling (extreme temperature and humidity fluctuations), with no evidence of significant deterioration of the material. Despite these advantages of the corn cob granules, the concrete produced with the corn cob granules exhibited higher water absorption than typical expanded clay concrete due to capillarity. The compressive strength of the corn cob concrete after 50 days was about 50% lower than that of the expanded clay concrete [101].

In the study by Helepciuc et al. [102], corn cob aggregates were incorporated into concrete mixes in three forms: untreated granules, granules treated with a sodium silicate solution, and granules treated with a more concentrated sodium silicate solution. The corn cob aggregates replaced 50% of the volume of the conventional aggregates (sand and stone). In the study, the density, compressive strength, tensile strength, flexural strength, and splitting strength of the resulting lightweight concrete were experimentally evaluated and compared with those of the reference concrete with natural aggregates. The results revealed significant differences between the reference concrete and the concrete produced with untreated corn cobs, but the mechanical properties were significantly improved by treating the corn cobs with sodium silicate. In conclusion, replacing natural aggregates with granulated corncobs results in lightweight concrete suitable for non-structural applications.

**Figure 27:**
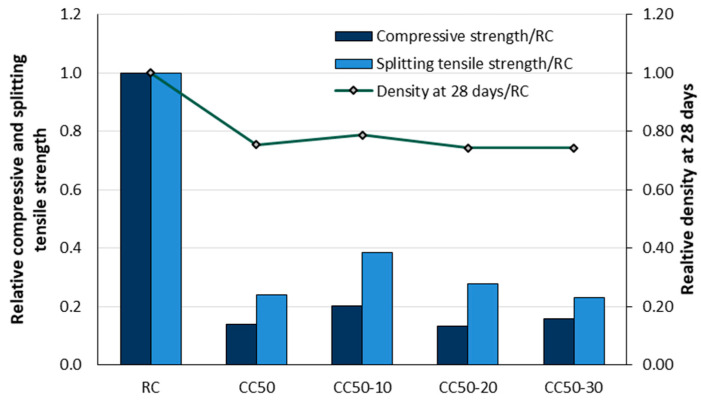
Relative compressive and splitting tensile strength (primary vertical axis) and relative density at 28 days (secondary vertical axis) of concrete with partial replacement of aggregate volume by corn cobs [102].

In another study by the same authors [102], the effects of replacing cement with fly ash at 0, 10, 20, and 30% by volume and replacing 50% of the volume of mineral aggregate with corn cobs were investigated. Fly ash improved the compressive strength in corn cob concrete mixtures with 10% (CC50-10) and 30% (CC50-30) replacement (Figure 27) and tensile splitting strength in mixtures with 10% (CC50-10) and 20% (CC50-20) replacement. However, these values remained significantly lower than those of the reference concrete (RC). The addition of corn cob aggregate resulted in a considerable reduction in concrete density, with around 25% reduction compared to the reference concrete.

**Figure 28:**
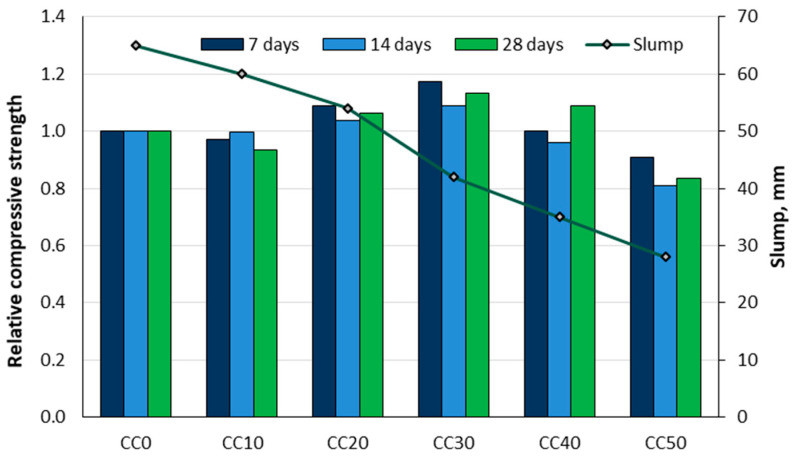
Relative compressive strength (primary vertical axis) and workability—slump (secondary vertical axis)—of concrete with partial replacement of aggregate volume by corn cobs [23].

Polat [103] investigated the potential use of corn cobs in the production of lightweight concrete. Four different mixtures composed of sand, ground corn cob, cement, and water were prepared, and the unit weight, thermal transmittance coefficient, and 28-day compressive strength were determined. The value of the 28-day compressive strength was found to be between 0.14 and 5.52 MPa, the heat transmissibility coefficient between 0.19 and 0.35 Kcal/m∙h∙°C, and the unit weight of the samples between 800 and 1520 kg/m^3^. Gradinari et al. [104] replaced mineral aggregates with crushed corn cobs in 20, 35, 50, 65, and 80% volume ratios. This concrete was analysed for density, compressive strength, splitting tensile strength, resistance to repeated freeze–thaw cycles, modulus of elasticity, and thermal conductivity. The corn cob aggregates reduced the compressive strength of the test specimens. For example, a 50% replacement led to an 86% reduction in compressive strength. Khan et al. [23] used corn cobs as a partial replacement for coarse aggregate in concrete blocks. The aim was to find the optimum percentage of natural coarse aggregate replacement that would provide similar or higher strength and the same workability compared to the reference concrete. The concrete was made from cement, sand, coarse aggregates, corn cobs, and marble dust. The cubes were cast and tested for compressive strength after 7 days, 14 days, and 28 days (Figure 28). The higher percentage of corn cobs as a substitute for coarse aggregate had a negative effect on the workability of the concrete (lower slump and stiffer concrete), while the compressive strength increased when about 30% of the aggregate was replaced by corn cobs.

Despite the limited number of studies in which crushed corn cobs were used as a substitute for natural aggregates, certain conclusions can be drawn. Concrete specimens formulated with this substitute material generally exhibit very low compressive strengths, as evidenced by the data presented in Table 6. An exception is the study of Khan et al. [23], in which marble dust was added to the mixes in addition to crushed corn cobs.

### 3.8. Rice Husk

#### 3.8.1. Production and Properties of Rice Husk

**Figure 29:**
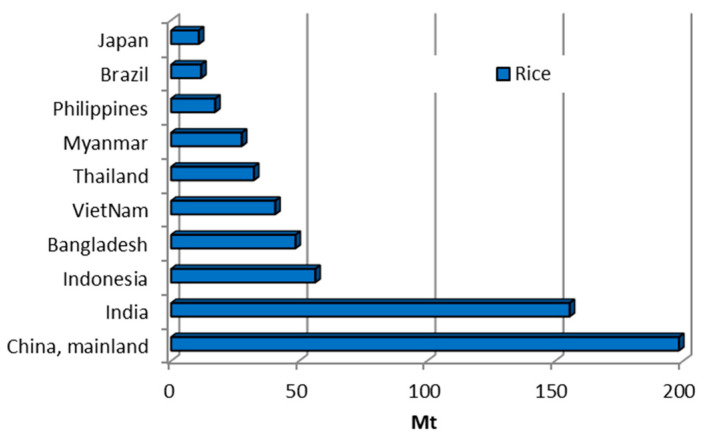
Production of rice. Top 10 producers (average in period 2002–2022); production data were taken from the website [10].

Rice (*Oryza sativa* L.) serves as a staple food for a substantial proportion of the global population and is cultivated on nearly all continents with the exception of Antarctica. Figure 29 presents the top 10 rice-producing countries from 2002 to 2022. Approximately 90.3% of global rice production is concentrated in Asia. Within the region, China and India are the largest producers, collectively accounting for over half of total global rice output [10]. Global rice production grew steadily during the study period, rising from 571.4 Mt in 2002 to 788.9 Mt in 2022.

When rice is processed, the outer layers, known as husks or bran, are removed from the grain. Rice husks typically constitute 20% to 24% of raw rice, with the average ratio of rice grain, husk, and straw being 1:0.25:1.25 [45]. Around 120 to 130 million tonnes of rice husks are produced every year. A portion of rice husks are repurposed as biofuel for heating and cooking applications, though the majority remain underutilised and are frequently abandoned in agricultural fields, contributing to waste management problems. The properties of rice husks vary depending on the type of rice, climatic conditions, and soil chemistry. Rice husks have an irregular, boat-like particle shape with a thickness of about 0.2 mm, a length of 8–10 mm, and a width of 2–3 mm. The packing density of rice husks is 122 kg/m^3^ [106]. According to Prusty et al. [19], rice husks have a hardness of 5–6 on the Mohs scale, and their dry bulk density ranges from 96 to 160 kg/m^3,^ while in [45], the bulk density of Japonica rice husk is reported as 380.54 kg/m^3^. The porosity of rice husks ranges from 63.64 to 73.23% [45], and their water absorption capacity can reach up to 130% [25]; see Table 1. The moisture content ranges from 4.6 [45] to 11.8% [25]. Rice husks and their ash are versatile materials with applications spanning numerous industries, offering significant potential for innovation and sustainability [19].

#### 3.8.2. Rise Husk as AWA

Rice husk has been used as a partial replacement for sand in concrete in experimental studies. Most previous studies have focused on using rice husk ash (RHA) and have shown that using RHA as a pozzolanic material can increase concrete materials’ compressive strength and durability [107,108,109]. However, fewer but significant studies investigated the direct use of rice husks as aggregates in concrete [46,109,110,111,112,113,114]. Salas et al. [109] used untreated rice husks and rice husks treated in a 5% lime solution. The volume of gravel and sand in normal concrete with low cement content (230 kg/m^3^) was gradually replaced by rice husks in proportions of 10, 20, 40, 60, and 80% for untreated rice husks and 5, 10, 20, and 40% for treated rice husks. The results showed that the treatment with rice husks significantly improved the compressive strength of the concrete. In addition, the authors found that untreated rice husks, when exposed to humid storage conditions, induced organic efflorescence in concrete specimens. Consequently, rice husk treatment effectively eliminated organic efflorescence.

**Figure 30:**
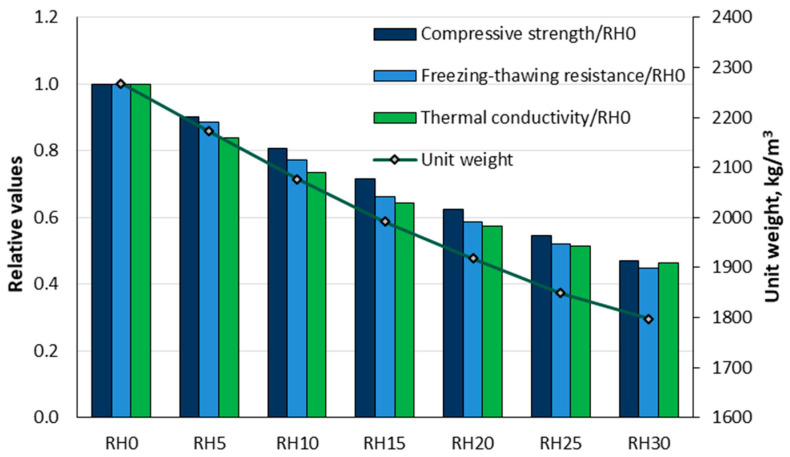
Relative values of compressive strength, freezing–thawing resistance, and thermal conductivity (primary vertical axis) and unit weight (secondary vertical axis) of concrete with partial replacement of aggregate volume by rice husk [110].

Sisman et al. [110] investigated the effects of adding rice husks to the regular aggregate at different volume proportions (5, 10, 15, 20, 25, and 30%). Figure 30 illustrates the effects of rice husk addition on the unit weight and the relative compressive strength values after 28 days, compressive strength after freeze–thaw cycles, and thermal conductivity of the test specimens. The test results showed that for the RH30 mixture (30% rice husk substitution), most properties were slightly over 50%, except for the unit weight, which decreased by approximately 20%. The compressive strengths of the concrete ranged from 17.6 to 37.5 MPa, whereas the unit weights varied between 1797 and 2268 kg/m^3^. All concrete samples demonstrated resistance, with water absorption values below 5.5%. In addition, the thermal conductivity values were between 1.53 and 0.79 W/mK. In summary, rice husks demonstrate potential as a material for lightweight concrete due to their strength, resistance, and insulating properties, making them suitable for use in agricultural buildings.

**Figure 31:**
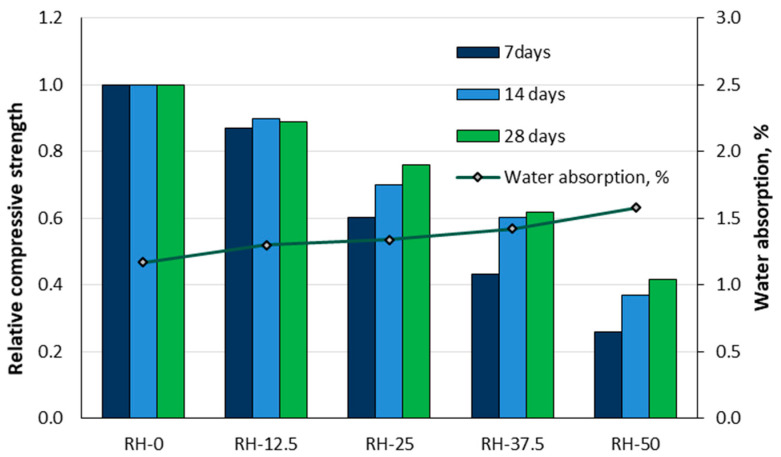
Relative compressive strength at 7, 14, and 28 days (primary vertical axis) and water absorption (secondary vertical axis) of concrete with partial replacement of fine aggregate volume by rice husk [46].

Akinwumi et al. [46] conducted research on the effects of partially replacing sand volume with rice husks in concrete. Laboratory tests were performed to evaluate the workability, air content, compressive strength, and water absorption properties of concrete with different proportions of rice husk. The rice husks were used as a substitute for sand in the following percentages: 0% (RH-0), 12.5% (RH-12.5), 25% (RH-25), 37.5% (RH-37.5), and 50% (RH-50). The results showed that the workability of the concrete improved with increasing rice husk content. However, the unit weight and compressive strength decreased with increasing rice husk content, whereas the water absorption and air content increased, as illustrated in Figure 31. The RH-50 mixture exhibited a 35% increase in water absorption, and the 28-day compressive strength decreased by 58.2% compared to the control concrete (RH-0). It is recommended to limit the replacement of rice husks in concrete for load-bearing applications to a maximum of 12.5.

Winarno [113] explored the use of rice husks in producing concrete blocks using a mixture of cement, aggregates, and rice husks. The study evaluated seven distinct cement-to-rice husk weight ratios ranging from 0.67 to 2.00, with a fixed water–cement ratio of 0.4. The specimens were tested for their 28-day strength. The results showed that higher proportions of rice husks led to lower strength. The optimal cement-to-rice husk weight ratio was identified as 1.34.

Chabi et al. [114] used rice husks as aggregates in a cement matrix to produce lightweight concretes for construction materials. Their research focused on the compatibility of cement with rice husks and the resulting concrete’s physical, mechanical, and thermal properties. Both the compressive and shear strength were determined in the study. In particular, the shear behaviour tests showed that the rice husk composites exhibited good shear behaviour, achieving shear strength up to 27% of the compressive strength, whereas the normal concrete exhibited very low shear strength.

Amantino et al. [26] concluded that using rice husk (RH) as a partial substitute for mineral fine aggregate in AWAC has shown promising results, particularly in reducing density and enhancing thermal performance compared to conventional concrete without AWAs. Specifically, incorporating 5% and 10% rice husk led to a decrease in compressive strength after six months, aligning with the expected outcomes for such material modifications.

Table 7 summarises the experimental results from the studies reviewed in this paper. Although rice husk ash is more commonly used in concrete, the studies demonstrate that rice husks themselves can also serve as a partial substitute for natural aggregates. However, rice husks tend to reduce the workability of concrete, as their coarse, fibrous texture and irregular shape increase internal friction. Concrete made from rice husks generally has a lower compressive strength than conventional concrete. However, research indicates that pretreatment of the husks can result in higher compressive strengths of the concrete samples. Rice husks possess good insulating properties which enhance the thermal performance of concrete, making them advantageous for energy-efficient building designs.

### 3.9. Sunflower Seed Hulls

#### 3.9.1. Production and Properties of Sunflower Seed Hulls

**Figure 32:**
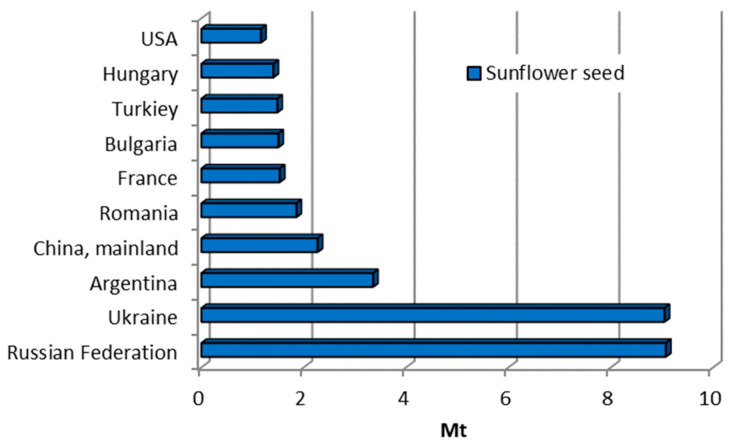
Production of sunflower seed. Top 10 producers (average in period 2002–2022); production data were taken from the website [10].

Sunflower (*Helianthus annuus*) is one of the most extensively cultivated oil crops worldwide. In 2022, global sunflower seed production reached 54.29 million tonnes, with Russia and Ukraine collectively accounting for approximately half of worldwide output, as illustrated in Figure 32.

**Figure 33:**
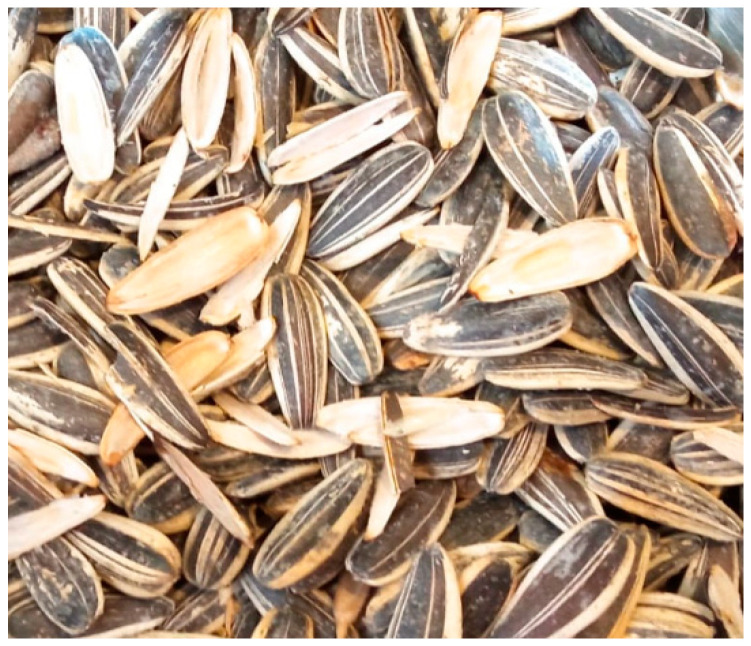
Sunflower hulls.

Sunflower seed hulls (shells), a significant by-product of the edible oil industry, account for approximately 20–30% of the mass of the processed seeds (Figure 33). In oil extraction plants, these hulls are often utilised as a heat source through combustion. As they are mainly composed of cellulose, sunflower seed hulls are a promising alternative biomass source for energy production and other industrial applications and offer opportunities for sustainable waste management. The chemical composition of sunflower seed hulls is characterised by a high crude fibre content of 58.73%, together with 4.63% ash, 30.74% carbohydrates, 2.28% protein, and 3.62% fat [115]. A study examining sunflower hulls subjected to palletisation [30] reported a moisture content of 9.61% and a bulk density of 139 kg/m^3^, as detailed in Table 1.

#### 3.9.2. Sunflower Seed Hulls as AWA

**Figure 34:**
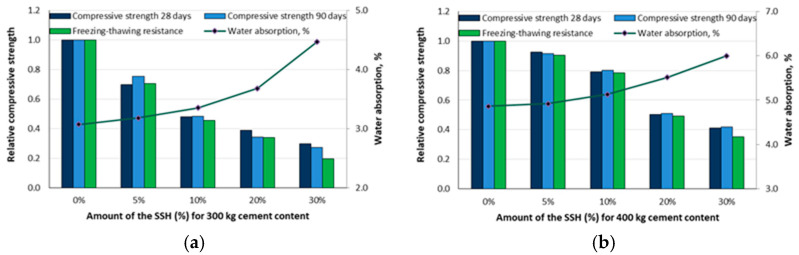
Relative compressive strength at 28 and 90 days, freezing–thawing resistance (primary vertical axis), and water absorption (secondary vertical axis) of concrete with partial replacement of aggregate volume by sunflower seed hulls for cement content in concrete of (**a**) 300 kg and (**b**) 400 kg [116].

The effects of sunflower seed hulls as aggregate replacement material on concrete properties were investigated by Sisman and Gezer [116] by adding different volumes of sunflower seed hulls (5, 10, 20, and 30%) in concretes with 300 and 400 kg of cement content. The mixtures achieved 28-day compressive strengths from 34.42 MPa to 10.20 MPa (300 kg cement dosage) and from 51.39 MPa to 21.08 MPa (400 kg cement dosage). Replacing aggregates with sunflower seed hulls significantly reduced the concrete compressive strength after 28 and 90 days and the freeze–thaw resistance while increasing the water absorption rate, as illustrated in Figure 34. The concrete specimens, excluding those containing 20% and 30% replacement in concrete with 300 kg of cement, were categorized as structural lightweight concrete based on their unit weight and compressive strength, whereas the concrete produced with 20% and 30% sunflower seed hulls was designated for use only as insulation concrete. The authors concluded that the samples tested and produced are particularly suitable for use in agricultural buildings [116].

Popa et al. [117] created an innovative finish/protection product for gypsum board surfaces using sunflower seed husks, which were incorporated into a finish/protection coating with good adhesion to the substrate and good thermal insulation characteristics.

## 4. Conclusions

The incorporation of agro-waste as a substitute material in concrete presents a highly promising solution to several pressing challenges in the construction industry, including the depletion of natural resources, high carbon dioxide emissions, and the accumulation of waste. Agricultural waste such as coconut shells, rice husks, palm kernel shells, crushed corn cobs, and various fruit seeds, shells, and pits possess physical and mechanical properties that allow for their use for partial or complete replacement of conventional aggregates. A growing body of research highlights that utilising these waste materials in concrete not only enhances sustainability but also significantly reduces the environmental footprint of the construction sector, contributing to the advancement of eco-friendly building materials.

From a technical standpoint, the use of agro-waste in concrete mixtures as an AWA offers a range of benefits. These include enhanced thermal and acoustic insulation properties. Furthermore, the relatively low density of many agro-waste aggregates reduces the overall weight of concrete, resulting in tangible economic advantages in transportation and on-site handling. Despite these advantages, several challenges must be addressed to fully realize the potential of agro-waste in concrete. Chief among them is the variability in the composition of agro-waste, which is influenced by geographical, climatic, and processing differences. This variability can affect the consistency, performance, and reliability of the final concrete product.

Another significant concern is the presence of organic impurities in untreated AWAs, which can interfere with the hydration process and setting time of cement. To address this issue, alkaline pretreatment of AWAs is recommended. This process not only removes residual organics from the surface of the waste material but also enhances the interfacial bond between the aggregate and the cement paste, improving the overall integrity of the concrete. These technical and chemical considerations underscore the importance of further research and the establishment of standardized treatment and processing protocols to ensure consistent, safe, and cost-effective use of agro-waste in construction.

Beyond its technical and environmental merits, the integration of AWAs into concrete production also presents notable socio-economic benefits. By creating value-added pathways for agricultural waste, this practice can generate additional income for farmers and stimulate business opportunities for industries engaged in waste collection and processing. It fosters job creation in the recycling and green construction sectors while simultaneously reducing material costs for builders increasingly seeking sustainable and affordable alternatives. Moreover, this approach encourages technological innovation and drives the development of new methods and products within the construction industry.

In conclusion, the use of AWAs in concrete production represents a holistic and forward-looking strategy for advancing sustainability in the built environment. It addresses critical ecological concerns, offers practical engineering solutions, and supports inclusive economic development. The systematic integration of agricultural residues into construction materials not only contributes to waste reduction and resource conservation but also aligns closely with global sustainable development goals. By embracing such circular practices, the construction industry can significantly reduce its environmental impact and help preserve climate stability and ecological health for future generations.

## Figures and Tables

**Table 1:** Properties of pits and shells as agro-waste aggregate (AWA) as well as crushed granite and river sand as conventional aggregate [4,18,19,20,21,22,23,24,25,26,27,28,29,30,31,32,33,34,35,36,37,38,39,40,41,42,43,44,45,46].

	Specific Gravity	Loose Bulk Density (kg/m^3^)	Compacted Bulk Density (kg/m^3^)	Bulk Density (kg/m^3^)	Water Absorption, %,	Moisture Content, %	Crushing Value, %	Impact Value, %	Los Angeles Abrasion Value, %	Flakiness Index, %	Shell Thickness, mm	Porosity, %
Palm kernel shells	1.02–1.62	500–600	596–685	-	9–33	4.35–15	0.2–10	2.03–8	3–5.70	65	0.5–8	28–37
Coconut shells	1.05–1.74	476–550	593–800	-	20–25	4–5.2	2–3	7–8.5	1.4–1.65	-	0.15–8	19
Hazelnut shell	-	550.50	-	-	-	14.38	-	-	-	-	0.95–2.41	-
Groundnuts (peanut) shell	0.3	-	-	254.55	-	6.39	-	-	-	-	-	-
Pistachios shell	0.12	-	-	286.9	-	4.04	-	-	-	-	-	-
Cherry pit	-	-	-	472.88	-	9.30	-	-	-	-	-	-
Peach shell	1.26–1.28	-	-	536–556	15.2–16.7	4.2	-	1.95	6–6.21	41–48	-	-
Apricot shell	1.42–1.44	-	-	600–630	10.9–13.8	2.8	-	2.95	6.82–7.83	48–56	-	-
Date seed	1.13–1.39	462	526	-	8.1–36	23	7.1	6.83–22	5.1			19.5
Grape seed	-	-	-	469.3–546.3	31	12.26–24.61	-	-	-	-	-	52.87–55.67
Maize (corn) cob	0.957	-	-	170–298	327	4.51–6.38	-	-	-	-	-	46.54–67.93
Rice husk	0.17	-	-	96–380.5	112–130	4.6–11.8	-	-	-	-	0.2	63.64–73.23
Sunflower seed	-	-	-	139	-	9.61	-	-	-	-		-
Crushed granite	2.60–2.70	1300	1420–1470	-	<1	-	20	13–17	24	-	25	-
River sand	2.56–2.65	-	-	1538–1680	1.2	-	-	-	-	-	-	0.04

**Table 2:** Mechanical properties of concrete with the addition of palm kernel shell (PKS) as a replacement for coarse aggregate.

Authors	Year	Replacement Percentage	w/c Ratio	Compressive Strength Range (MPa)
Mannan and Ganapathy [50]	2004	PKS as 100% coarse aggregate volume	0.41	12.61–30.05 MPa curing condition: full water 12.61–24.74 MPa curing condition: partial water 11.51–29.69 MPa curing condition: plastic film
Ifeanyi et al. [55]	2023	PKS as 10%, 20%, and 30% replacement for coarse aggregate volume	-	13.4–34.1 MPa
Khankhaje et al. [56]	2016	0–75% PKS as coarse aggregate + 25–75% limestone (by mass); PKS grain size 4.75–6.30 mm and 6.30–9.5 mm	0.32	6–12 MPa
Azunna [57]	2019	PKS as 10% and 25% replacement for coarse aggregate mass	0.50	4.78 and 4.44 MPa
Ngagoum et al. [58]	2020	PKS as 25, 50, 75, and 100% aggregate volume	0.59, 0.64, 0.68, 0.73	5.37–19.66 MPa
Shafigh et al. [59]	2011	PKS as 100% coarse aggregate volume	0.30, 0.34, 0.35, 0.44	34–53 MPa
Mo et al. [60]	2016	PKS as 100% coarse aggregate and 20–60% GGGS as replacement for cement	0.40	24.4–46.0 MPa za PKS concrete water curing 12.5–20.2 MPa PKS concrete air curing
Olanipekun et al. [61]	2005	PKS as 25, 50, 75, and 100% aggregate volume	0.5, 0.75	17.5–24.9 MPa
Alengaram et al. [62]	2010	PKS as 100% coarse aggregate, 5% fly ash, and 10% silica fume	0.35	About 26 MPa
Gibigaye et al. [63]	2016	PKS/fine aggregate mass in range 0.4–0.75	0.45	18.63 MPa
Danso and Appiah-Agyei [64]	2021	PKS as 100% coarse aggregate mass	0.60	8.8–10.2 MPa

**Table 3:** Mechanical properties of concrete with the addition of coconut shell (CS) as a replacement for coarse aggregate.

Authors	Year	Replacement Percentage	w/c Ratio	Compressive Strength Range (MPa)
Olanipekun et al. [61]	2006	25, 50, 75, 100% CS as a replacement for coarse aggregate volume	0.5, 0.75	16–27.5 MPa
Yerramala and Ramachandrudu [67]	2012	10, 15, 20% CS as a replacement for coarse aggregate mass	0.60	9.33–13.56 MPa
Gunasekaran et al. [68]	2008	100% CS as coarse aggregate	0.42	19.1 MPa
Osei [69]	2013	20, 30, 40, 50%, and 100% CS as a replacement for coarse aggregate volume	0.6	9.29–19.7 MPa
Kanojia and Jain [70]	2017	10, 20, 30, 40% CS as a replacement for coarse aggregate volume	0.55	22.2–25.6 MPa
Azunna et al. [71]	2018	10, 20, 30% CS as a replacement for fine and coarse aggregate mass	0.45	25.21–38.37 MPa
Gunasekaran et al. [72]	2011	100% CS as coarse aggregate	0.42–0.72	4.95–26.7 MPa
Deepak et al. [73]	2015	10, 20, 30, 40, 50% CS as a replacement for coarse aggregate mass	0.45	18.4–33 MPa
Rajendran and Abdul Rahman [74]	2022	10, 20, 30% CS as a replacement for coarse aggregate mass	0.50	33.16–37.50 MPa
Manaloto [75]	2023	2.5, 5, 7.5, 10, 15, 20, 100% CS as a replacement for coarse aggregate mass	0.647	16–20.69 MPa

**Table 4:** Mechanical properties of concrete with the addition of stone fruit shells and pits: cherry pits (CPs), peach shells (PSs), and apricot shells (ASs) as a replacement for aggregate.

Authors	Year	Replacement Percentage	w/c Ratio	Compressive Strength Range (MPa)
Wu et al. [27]	2018	100% crushed PS and AS as coarse aggregate	0.40	19.6 and 26.9 MPa
Netinger Grubeša et al. [34]	2022	volume of coarse aggregate fraction was replaced with cherry pits untreated and treated with 2.5 and 5% NaOH	0.40	14.46–19.64 MPa
D’Eusanio et al. [36]	2023	100% PS and AS as coarse aggregate	0.40–0.45	1–4 MPa 2.8–7 MPa
Wu et al. [40]	2018	12.5, 25, 37.5, 50% crushed PS and AS as a replacement for coarse and fine aggregate volume	0.35	33.5–46.2 MPa
Wu et al. [43]	2020	25, 50, 75, 100% PS as a replacement for normal weight aggregate volume	0.35	25.7–38.7 MPa
Zwicky [83]	2020	12% GS, 25% CP, 57% GSCP, and 37% GSCP as a replacement for natural aggregate volume	-	12.9–18 MPa
Wu et al. [87]	2018	100% PS as coarse aggregate	0.33	24.6 MPa
Yildiz et al. [88]	2012	5–40% replacement of limestone aggregate volume with AS	0.55	14.92–32.05 MPa

**Table 5:** Mechanical properties of concrete with the addition of date seeds (DSs) as a replacement for coarse aggregate.

Authors	Year	Replacement Percentage	w/c Ratio	Compressive Strength Range (MPa)
Adefemi et al. [20]	2013	25, 50, and 75% DS as a replacement for coarse aggregate mass	0.6 and 0.65	16–23.44 MPa 9–14.4 MPa
Ahmed et al. [90]	2020	2, 3, and 4% DS as a replacement for coarse aggregate mass	0.50	11.76–13.83 MPa
Palh et al. [91]	2021	2, 3, and 4% DS as a replacement for coarse aggregate volume	0.6	13.63–20.87 MPa
Yusuf et al. [92]	2021	5, 10, 15, and 20% DS as a replacement for coarse aggregate mass	0.50	18.1–20.3 MPa
Sarathkumar et al. [94]	2022	10 to 20% of DS and 1.5 to 2.5% of tamarind seed as a partial replacement for fine aggregate mass	0.45	26.40–28.43 MPa

**Table 6:** Mechanical properties of concrete with the addition of corn cobs (CCs) as a replacement for coarse aggregate.

Authors	Year	Replacement Percentage	w/c Ratio	Compressive Strength Range (MPa)
Khan et al. [23]	2022	10, 20, 30, 40, and 50% CC as a replacement for coarse aggregate volume	0.50	21.6–24.25
Pinto et al. [100]	2012	100% CC as coarse aggregate	-	0.39–0.51
Grădinaru et al. [105]	2017	50% CC as a replacement for coarse aggregate volume	0.43	0.27–10.21
Helepciuc et al. [102]	2018	50% CC as a replacement for coarse aggregate volume and 10, 20, and 30% fly ash as a replacement of cement	-	3.35–5.10
Polat [103]	2021	100% CC as coarse aggregate	0.44–0.77	0.14–5.52
Gradinaru et al. [104]	2021	20, 35, 50, 65, and 80% CC as a replacement for coarse aggregate volume	0.50	3.04–9.79

**Table 7:** Mechanical properties of concrete with the addition of rice husk (RH) as a replacement for aggregate.

Authors	Year	Replacement Percentage	w/c Ratio	Compressive Strength Range (MPa)
Amantino et al. [26]	2022	5 and 10% RH as a replacement for natural sand volume	0.58	15–19 MPa
Akinwumi et al. [46]	2016	12.5, 25, 37.5, and 50% RH as a replacement for sand volume	0.50	8.8–18.5 MPa
Salas et al. [109]	1986	80, 60, 40, 20, and 10% untreated RH as a replacement for aggregate volume and 40, 20, 10, and 5% treated RH as a replacement for aggregate volume	0.71	0.16–14.96 MPa 0.36–0.66 MPa
Sisman et al. [110]	2011	5, 10, 15, 20, 25, and 30% RH as replacement for aggregate volume	0.60	17.6–37.5 MPa
Winarno et al. [113]	2019	100% RH as aggregate	0.40	1.1–1.9 MPa
Chabi et al. [114]	2020	100% RH as fine aggregate	0.30–0.45	1.93–15.63 MPa

## Data Availability

Data sharing is not applicable. No new data were created or analyzed in this study.
